# Directly and Simultaneously Expressing Absolute and Relative Treatment Effects in Medical Data Models and Applications

**DOI:** 10.3390/e23111517

**Published:** 2021-11-15

**Authors:** Haoyang Teng, Zhengjun Zhang

**Affiliations:** 1 Department of Mathematics and Statistics, Arkansas State University, P.O. Box 70, Jonesboro, AR 72467, USA; 2 Department of Statistics, University of Wisconsin-Madison, 1300 University Ave, Madison, WI 53706, USA; zjz@stat.wisc.edu

**Keywords:** asymptotics, enhanced logistic regression, estimator consistency, interpretability, precision medicine, predictability

## Abstract

Logistic regression is widely used in the analysis of medical data with binary outcomes to study treatment effects through (absolute) treatment effect parameters in the models. However, the indicative parameters of relative treatment effects are not introduced in logistic regression models, which can be a severe problem in efficiently modeling treatment effects and lead to the wrong conclusions with regard to treatment effects. This paper introduces a new enhanced logistic regression model that offers a new way of studying treatment effects by measuring the relative changes in the treatment effects and also incorporates the way in which logistic regression models the treatment effects. The new model, called the Absolute and Relative Treatment Effects (AbRelaTEs) model, is viewed as a generalization of logistic regression and an enhanced model with increased flexibility, interpretability, and applicability in real data applications than the logistic regression. The AbRelaTEs model is capable of modeling significant treatment effects via an absolute or relative or both ways. The new model can be easily implemented using statistical software, with the logistic regression model being treated as a special case. As a result, the classical logistic regression models can be replaced by the AbRelaTEs model to gain greater applicability and have a new benchmark model for more efficiently studying treatment effects in clinical trials, economic developments, and many applied areas. Moreover, the estimators of the coefficients are consistent and asymptotically normal under regularity conditions. In both simulation and real data applications, the model provides both significant and more meaningful results.

## 1. Introduction

Studying treatment effects is central in clinical trials and epidemiology. When response variables are dichotomous, numerous applications of the logistic regression model can be found in the literature. Using the logistic regression model in the analysis of the medical data allows the researchers to understand and estimate the effects of the explanatory variables on the response variable, control the confounding factors and study the interaction effects. The purpose of the analysis using the logistic regression is to identify risk factors that are associated with the response variable of interests and the variables (confounder) that influence the effect of exposure on disease and the risk factors. For instance, if the primary goal is to measure the association between physical inactivity and heart disease with age being a confounding factor, the logistic regression is not only useful to model dichotomous variables (e.g., the values of 0 and 1 represent the status of heart disease, respectively), but it can also be used to explain the effects of physical inactivity on heart disease while controlling for the age variable. Odds ratio, which is often used for interpretations in the logistic regression model, is adjusted to account for other covariates (including confounders). Other applications can be found in genetics, clinical trials, or any studies that involve treatment groups. This statistical model has been a benchmark model due to its easy computability, interpretability, predictability, and stability (CIPS).

Logistic regression is also widely used in classifications, e.g., in cancer [1], diabetes [2], and osteoarthritis [3] among numerous literature publications. Due to the desirable CIPS properties, logistic regression is often used as a baseline/benchmark model in popular machine learning to perform classification, including via Support Vector Machine (SVM) [4] and Naive Bayes Classifier [5]. Applications using machine learning models can be found in acute coronary syndromes [6], heart failure [7], pancreatic cancer [8], text mining [9], COVID-19 and seven subtypes [10], etc.

The studies of logistic regression and related models have been drawing much attention in the literature. For instance, as the number of predictor dimensions increases and exceeds the sample size, direct estimation using the logistic regression model may fail because the matrix inversion can be a problem due to the matrix being singular. In addition, issues such as numerical problems lead to poor convergence, overfitting and low predictive power [11,12]. Regularization is often used to handle high-dimensional data. Popular penalty functions include but are not limited to the least absolute shrinkage and selection operator (Lasso) [13], the smoothly clipped absolute deviation (SCAD) [14] and the minimax concave penalty (MCP) [15]. On the other hand, there have been many model developments in the handling of semi-continuous data in recent years where the response data consist of a substantial portion of single value and positive values. In handling such data, a two-part model was proposed, which handles a combination of binary and continuous data. Since the logistic regression model, which preserves many desirable properties, is suitable to model the binary part, it is included as part of the model. The model development and discussions can be found in recent studies [16,17,18].

In this paper, we focused on developing a more general logistic regression model. Our paper’s contributions to the literature can be concluded in three-fold: (1) The Absolute and Relative Treatment Effects (AbRelaTEs or Abrelates) are directly, explicitly, and simultaneously introduced in our proposed enhanced logistic regression. The AbRelaTEs model incorporates how treatment effects are modeled in the classical logistic regression (absolute treatment effect) and offers a different way of modeling the effects (relative treatment effect). In the model, the absolute treatment effect incrementally measures the treatment effects, while the relative treatment effect accounts for the proportion change in the treatment effects. Additionally, the new model unifies the logistic regression model in a new framework. Parameter estimation can be easily performed using software packages, with the classical logistic regression model being treated as a particular case. (2) The interpretations can be made in two ways, via “between-group” and “within-group” treatments. The former considers all covariate information (attributes) of each patient/participant, which can be regarded as an individualized effect. If the individualized effect is better for a patient/participant in the treatment group than the control group, the treatment is suitable for the patient. The treatment can also be recommended for other patients with similar attributes, making it a potential precision medicine. The latter, on the other hand, individually interprets the effects of each predictor. (3) Simulation examples show that the classical logistic regression will fail to model data when a relative treatment effect exists. In addition, the statistical model might fail to capture the absolute treatment effect if the relative treatment effect exists, resulting in researchers being misled into believing that the treatments are not significant. The AbRelaTEs model, which offers another way of modeling the treatment effects, captures the effects via an absolute or relative or even both. These were shown to be possible as explored using four real datasets. The AbRelaTEs model can be viewed as a new benchmark model for randomized controlled trial studies.

This paper is organized as follows. In Section 2.1, we will present the classical logistic regression model. We then introduce the Absolute and Relative Treatment Effects model and discuss the interpretations in Section 2.2. In Section 3, we will discuss the relative effect term’s estimation procedures and other coefficient parameters of the new model. Moreover, asymptotic theories such as the model’s consistency and asymptotic normality are presented in the same section. The new model’s computational procedures will be discussed in Section 4 and simulation examples and discussions will be presented in Section 4. Furthermore, four real data examples will be explored and discussed in detail in Section 5. Finally, the concluding remarks are given in Section 6. Technical arguments and additional simulation results are presented in the Appendices.

## 2. Logistic Regression and the Enhanced Absolute and Relative Treatment Effects Model

In this section, we will first present the logistic regression model and then introduce the AbRelaTEs model. We will deliver the features and interpretations of the AbRelaTEs model in a more general aspect, and more detailed interpretations will be discussed using the real data examples in the latter sections.

### 2.1. Logistic Regression

Before introducing our model, we provide an overview of the ordinary logistic regression model commonly used to model data with binary outcomes. Due to its easy application and high interpretability, the model is often used to analyze data in various fields. One common application can be found in randomized controlled trials to investigate whether the treatment effects are significant in explaining the outcomes. If the treatment effects are significant, meaningful interpretations of the treatment effects and other covariates are often made in the forms of an odds ratio and relative risk.

We now describe the classical logistic regression model in a randomized controlled trial setting. Suppose we consider *g* treatment groups. Throughout this paper, the *g*-th treatment group is considered a control group. In addition, the term “treatment group” excludes the control group to distinguish and make comparisons with the control group throughout this paper. In the *j*-th group, we have $n_{j}$ patients with total patients being *n*. Let $Y_{ij}$ be the binary response (0 or 1) of *i*-th patient in treatment group *j*. Let μ be a constant and $\tau_{j}$ be the treatment effect of *j*-th treatment level. Let $X_{ij}$ be a p×1 covariate vector where *p* is the number of predictors and β is the corresponding p×1 coefficient vector. Denote by $\pi_{ij}$ the probability $P(Y_{ij}=1|X_{ij})$ for $i=1,2,...,n_{j}$ and j=1,2,...,g. The classical logistic regression model is given by

(1) $$\mathrm{logit}\left(\pi_{ij}\right)=\log\left(\frac{\pi_{ij}}{1-\pi_{ij}}\right)=\mu +\tau_{j}+X_{ij}^{\prime }\beta ,$$

where $i=1,2,...,n_{j}$ and j=1,2,...,g. The observations are i.i.d. samples $\left(Y_{ij},X_{ij}\right)$ for $i=1,2,...,n_{j}$, j=1,2,...,g and $Y_{ij}|X_{ij}∼\mathrm{Bernoulli}\left(\pi_{ij}\right)$. We note that the model (1) has been widely used as a benchmark model in many classification problems and treatment effects analyses in medical data. In many real applications, if the (absolute) treatment effects $\tau_{j}$s in model (1) are found to be significant, the treatment groups can then be recommended to be practiced or adopted by the general public. If the treatment effects result insignificant, the classical logistic regression is not capable of measuring the treatment effects and is deemed to be insufficient to model treatment effects for some clinical trials, but is actually effective. If the treatment effects are tested and result not significant, the logistic regression model not only fails to detect any overall treatment effect but it also has a low predictive power. Furthermore, the treatment effect $\tau_{j}$ in the classical logistic regression does not detect any individualized effect of the treatment groups. If the treatment group is found to be significant, it is highly questionable that the treatment group will be effective for all patients. It is of interest to many researchers whether the treatment groups can be further interpreted as precision medicine for specific groups of people with the same attributes or characteristics, which is also one of the aspects of this paper. In contrast to the approaches in the literature, we generalize the model (1) which preserves many desirable properties both theoretically and practically to serve the purposes mentioned above but with better and easier interpretations. We will present and discuss our model in the following subsection.

### 2.2. Logistic Regression with Absolute and Relative Treatment Effects

We will first introduce some additional notations and some motivations before presenting our model. In the literature, both absolute errors, e.g., |a−b|=|τ|, and relative errors, e.g., |(a−b)/b|=|δ| or a=(1+δ)b are useful and powerful measurements for studying changes between two variables *a* and *b*. In many applied scientific areas, relative changes are also regarded as an increasing rate or decreasing rate; e.g., in economics, we measure the gross domestic product (GDP) change using the rate; in finance and banking, the changes are also termed returns or interests. Without loss of generality, we shall call τ and $\tau_{j}$ absolute errors or absolute changes, and δ and $\delta_{j}$ relative errors or relative changes throughout the paper.

Motivated by the relative measurements, we propose a model that also considers the relative treatment effects of the treatment groups in addition to the absolute treatment effects $\tau_{j}$ of the treatment groups in model (1). Moreover, we also include important predictors in our model. Let $\delta_{j}$ be the relative treatment effect of the *j*-th treatment level. Then, our newly proposed model, the Absolute and Relative Treatment Effects (AbRelaTEs) Model, is given by

(2) $$\mathrm{logit}\left(\pi_{ij}\right)=\log\left(\frac{\pi_{ij}}{1-\pi_{ij}}\right)=\left(\mu +\tau_{j}+X_{ij}^{\prime }\beta \right)\left(1+\delta_{j}\right),$$

for $i=1,2,...,n_{j}$ and j=1,2,...,g. In our setting, the parameters μ, $\tau_{j}$ and β are similarly defined in the logistic regression model setting. Since the parameter $\delta_{j}$ in our model measures the relative effect of the treatments, the parameter can take any value between −1 and 1.

It is clear that model (2) will be reduced to model (1) when $\delta_{j}=0$ for all *j*, and that there is no relative treatment effect. We note that when model (1) is the true model, model (2) is also true since $\delta_{j}$ will be estimated to be 0. Furthermore, it is worth noting that the model (2) is the same as the classical logistic regression when no covariates $\left(X_{ij}\right)$ are available. On the other hand, if model (1) is not a correct/appropriate model for the analysis of a randomized controlled trial, model (2) is still applicable. Therefore, the AbRelaTEs model can serve as a new “benchmark" model for better applicability, more flexibility, and increased interpretability, which can be applied to many fields of medical research.

In our model setting, the term $1+\delta_{j}$, which will always be positive, can be viewed as a multiplier effect on the log-odds depending on the sign and magnitude of the estimated relative treatment effects of $\delta_{j}$. In other words, if the relative treatment effect is significant for a randomized controlled trial, there will be an additional multiplier effect on the log-odds for patients receiving treatments compared to the control group. The multiplier effect on the log-odds will depend on the estimated coefficients of the constant μ, absolute treatment effect $\tau_{j}$, and covariates $X_{ij}$. In fact, the multiplier effect is more interpretable by computing the overall magnitude and sign of the term $\mu +\tau_{j}+X_{ij}^{\prime }\beta$. Since the covariates $X_{ij}$ are usually the attributes or characteristics of a patient (e.g., weight, height, age, gender, etc.) in randomized controlled trials, the relative treatment effect will have a different impact for different patients if some attributes are continuous or a group of patients sharing similar attributes if the covariates are all discrete or categorical in a particular treatment group. For example, patients in a specific weight range and age group will benefit more from receiving the treatment than patients in other weight range and age groups. As a result, the relative treatment effect is the key to measure individualized treatment effects, and the model (2) can be viewed as a benchmark model dealing with precision treatments which can be seen as an advantage over the classical logistic regression model.

Model (2) can be expressed as

(3) $$\mathrm{logit}\left(\pi_{ij}\right)=\log\left(\frac{\pi_{ij}}{1-\pi_{ij}}\right)=\left(\mu +\tau_{j}^{*}+X_{ij}^{\prime }\beta_{j}^{*}\right),$$

for $i=1,2,...,n_{j}$ and j=1,2,...,g, where $\tau_{j}^{*}=\tau_{j}+\mu \delta_{j}+\tau_{j}\delta_{j}$ and $\beta_{j}^{*}=\beta \left(1+\delta_{j}\right)$. We note that the AbRelaTEs model is different from the classical logistic regression where the coefficient vector $\beta_{j}^{*}$ in Equation (3) depends on the treatment group and it is not the case for the classical logistic regression though the form resembles the classical logistic regression. In the model setup, the effects of the coefficient depend on the treatment groups of the patients. For patients receiving the treatments, the coefficient is $\beta (1+\delta_{j})$ for j=1,2,...,g−1 while the coefficient is β for patients in the control group. The coefficients are different for patients receiving different treatment. From the construction of the model, the AbRelaTEs model is different from the standard logistic regression model including interactions between variables. Furthermore, the AbRelaTEs model can also be expressed in the following form:

(4) $$\mathrm{logit}\left(\pi_{ij}\right)=\log\left(\frac{\pi_{ij}}{1-\pi_{ij}}\right)=\left(\mu +\tau_{j}^{*}+{\tilde{X}}_{ij}^{\prime }\beta \right),$$

for $i=1,2,...,n_{j}$ and j=1,2,...,g, where $\tau_{j}^{*}=\tau_{j}+\mu \delta_{j}+\tau_{j}\delta_{j}$ and ${\tilde{X}}_{ij}^{\prime }=X_{ij}^{\prime }\left(1+\delta_{j}\right)$.

At a first glance, model (4) looks like a classical logistic regression model. However, upon closer examination of ${\tilde{X}}_{ij}^{\prime }=X_{ij}^{\prime }\left(1+\delta_{j}\right)$, we see that within the *j*-th treatment, each component covariate has a multiplier of $1+\delta_{j}$, i.e., $|\delta_{j}|$ is the relative error of ${\tilde{X}}_{ij}^{\prime }$ to $X_{ij}^{\prime }$. Note that in $X_{ij}^{\prime }$, some components can be products of other component variables, i.e., interactions, which are also kept in ${\tilde{X}}_{ij}^{\prime }$. As a result, expressing the logistic regression model as the AbRelaTEs model clearly shows that $\delta_{j}$ is a relative treatment effect coefficient, and it should not be interpreted as an interaction effect between $\tau_{j}$ and the covariates.

In the classical logistic regression, after computing the odds ratios or relative risks, the treatment effects can be related to covariates. Conventionally, the interpretations can be made based on each treatment group’s effects and predictors using the coefficients’ magnitude and sign. However, the interpretations of the coefficients in our model are not as straightforward. The interpretations can be made in two ways which are “between-group" and “within-group” treatments. For the “within-group” treatment effect, each predictor’s effect on the log-odds is interpreted. In contrast, all covariates for each patient are considered for the “between-group” treatment effect. Whether or not a treatment group is suitable for all people or a particular subgroup of people depends on the interpretations of the “between” group treatment. If treatment is beneficial for an individual or a subgroup of people with similar attributes, the treatment is viewed as precision medicine.

To illustrate the concepts of the absolute and relative treatment effects, we consider the case of two treatment groups, i.e., g=2 with $\tau_{g}$ and $\delta_{g}$ being 0. Additionally, we also assume that the response variable is the event of a patient having a particular disease with the same attributes. We first consider the case where there is an absolute treatment effect without a relative treatment effect. The absolute change in the log odds between the treatments or log odds ratio of a patient contracting the disease is given as (j=1 when g=2)

(5) $$\begin{array}{cc} \log(\mathrm{Odds}(\mathrm{Treatment}))-\log(\mathrm{Odds}(\mathrm{Control})) & =\left(\mu +\tau_{j}+X_{ij}^{\prime }\beta \right)-\left(\mu +X_{ij}^{\prime }\beta \right)=\tau_{j}. \end{array}$$

Subsequently, we consider the case where there is a relative treatment effect without an absolute treatment effect. The change in the log odds can be measured in the following way:

(6) $$\begin{array}{cc} \frac{\log(\mathrm{Odds}(\mathrm{Treatment}))-\log(\mathrm{Odds}(\mathrm{Control}))}{\log(\mathrm{Odds}(\mathrm{Control}))} & =\frac{\left(\mu +X_{ij}^{\prime }\beta \right)\left(1+\delta_{j}\right)-\left(\mu +X_{ij}^{\prime }\beta \right)}{\left(\mu +X_{ij}^{\prime }\beta \right)}=\delta_{j}. \end{array}$$

It is worth noting that $\delta_{j}$ under the circumstances measures the relative change in the context of log odds. When considering both treatment effects, the interpretations and forms are not as straightforward. The absolute change in the log odds between the treatments or log odds ratio of a patient contracting the disease is given as (j=1 when g=2)

(7) $$\begin{array}{cc} \log(\mathrm{Odds}(\mathrm{Treatment}))-\log(\mathrm{Odds}(\mathrm{Control})) & =\left(\mu +\tau_{j}+X_{ij}^{\prime }\beta \right)\left(1+\delta_{j}\right)-\left(\mu +X_{ij}^{\prime }\beta \right)\\ \; & =\tau_{j}+\left(\mu +\tau_{j}+X_{ij}^{\prime }\beta \right)\delta_{j}. \end{array}$$

If there is no relative treatment effect ($\delta_{j}=0$), the log odds ratio computation only depends on the treatment effect $\tau_{j}$, which is the case of the classical logistic regression. When there is a relative treatment effect ($\delta_{j}\neq 0$), the log odds ratio of contracting the disease also depends on the attributes $X_{ij}^{\prime }$ of the patient. The treatment group will have varying changes depending on $\left(\mu +\tau_{j}+X_{ij}^{\prime }\beta \right)$, e.g., a larger decrease in the log odds ratio for some patients i=1,2,...,n of the same attributes and a smaller decrease for a certain group of people are possible. For example, if the effect of a particular treatment is more prominent for obese patients than patients with a normal body weight holding other attributes constant, this is reflected in the smaller log odds ratio for the former patients than the latter. As a result, the AbRelaTEs model is ideal for interpreting the treatment effect in the context of precision medicine for some patients i=1,2,...,n.

Furthermore, the relative treatment effect in the AbRelaTEs model can be better explained in the context of the percentage increase/decrease, ((a−b)/b), in the odds of contracting the disease in a particular treatment group as discussed at the beginning of the section. The relative change in the odds between the treatments is:

$$\begin{array}{cc} \frac{\mathrm{Odds}(\mathrm{Treatment})-\mathrm{Odds}(\mathrm{Control})}{\mathrm{Odds}(\mathrm{Control})} & =\frac{\exp\{\left(\mu +\tau_{j}+X_{ij}^{\prime }\beta \right)\left(1+\delta_{j}\right)\}}{\exp\{\mu +X_{ij}^{\prime }\beta \}}-1\\ \; & =\exp\left\{\tau_{j}\right\}\exp\left\{\left(\mu +\tau_{j}+X_{ij}^{\prime }\beta \right)\delta_{j}\right\}-1. \end{array}$$

Since the effect of $\tau_{j}$ is constant while the effect of $\delta_{j}$ is proportional based on the absolute change in log odds and relative change in odds, we name the effects of $\tau_{j}$ and $\delta_{j}$ the absolute and relative effects in this paper.

In addition, we present additional discussions on different settings of the parameter values of $\tau_{g}$ and $\delta_{g}$ which can be set differently under our model setting for g=2. The parameters for the control group effects are $\tau_{2}=-\tau_{1}$ and $\delta_{2}=-\delta_{1}$ using the constraints $\sum_{j=1}^{2}\tau_{j}=0$ and $\sum_{j=1}^{2}\delta_{j}=0$ by the convention. Using the example for Equation (5), the absolute change in the log odds between the treatments or log odds ratio of a patient contracting the disease is given as (j=1 when g=2):

(8) $$\begin{array}{cc} \log(\mathrm{Odds}(\mathrm{Treatment}))-\log(\mathrm{Odds}(\mathrm{Control})) & =\left(\mu +\tau_{j}+X_{ij}^{\prime }\beta \right)-\left(\mu -\tau_{j}+X_{ij}^{\prime }\beta \right)=2\tau_{j}. \end{array}$$

The relative change in log odds for the case of a relative treatment effect without an absolute treatment effect is given in the following:

(9) $$\begin{array}{cc} \frac{\log(\mathrm{Odds}(\mathrm{Treatment}))-\log(\mathrm{Odds}(\mathrm{Control}))}{\log(\mathrm{Odds}(\mathrm{Control}))} & =\frac{\left(\mu +X_{ij}^{\prime }\beta \right)\left(1+\delta_{j}\right)-\left(\mu +X_{ij}^{\prime }\beta \right)\left(1-\delta_{j}\right)}{\left(\mu +X_{ij}^{\prime }\beta \right)\left(1-\delta_{j}\right)}\\ \; & =\frac{2\delta_{j}}{1-\delta_{j}}. \end{array}$$

We first note that $\tau_{j}$ under this constraint can still be viewed as an absolute treatment effect. However, it can be seen from Equation (9) that the relative change in log odds is not equal to $\delta_{j}$ when g=2, as shown in Equation (6) without using the constraints. Even though this constrained setting does not affect the interpretability aspects of our model as we interpret the additional treatment effects (i.e., relative treatment effect) of our model for every individual discussed above, the impact of $\delta_{j}$ is not exactly relative when using the constraints. On a further note, the constraints can be applied to g>2 for the absolute treatment effects, but are not applicable for the relative treatment effects under our setting. For instance, we consider the case of g=3. If $\delta_{1}$ and $\delta_{2}$ take values of 0.5 and 0.7, respectively, then $\delta_{3}$ will take a value of −1.2 under the constraint which violates our model assumption on $\delta_{j}$ (i.e., $-1<\delta_{j}<1$ for j=1,2,...,g). For g=2, the constrained setting can be applied in our model setting for both $\tau_{j}$ and $\delta_{j}$. It cannot only be viewed as a special setting in our model framework but also provides more flexibility in modeling the treatment groups. For a more general framework g>2, we require that $\delta_{g}=0$ and the additional treatment effect is interpreted as a relative treatment effect on the baseline group which is the control group. Subsequently, the previous discussions for g=2 can be extended to g>2, which we will not further discuss in this paper.

### 2.3. Toy Example

**Figure 1:**
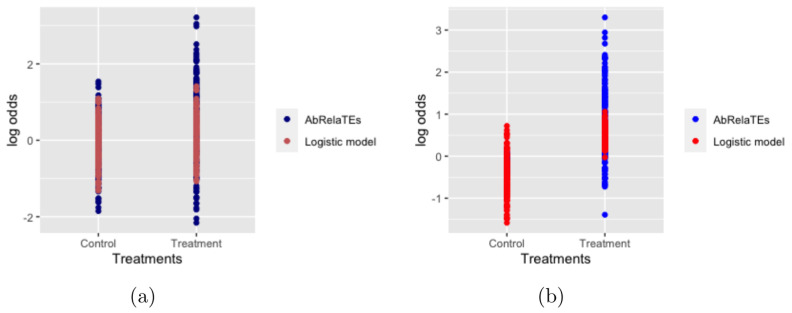
Log odds of two simulated examples are plotted for the AbRelaTEs model and logistic regression model. The simulated example in panel (**a**) considers treatment effects and covariate effects. The simulated example in panel (**b**) considers treatment effects, covariate effects and an interaction effect between the covariate and treatment.

In this subsection, we provide some toy examples to better understand the AbRelaTEs model. Two simulated examples from the AbRelaTEs model are presented in Figure 1 and compared to the logistic regression model. The simulated example in the left panel is simulated with two levels of treatments and two covariates and the simulated example in the right panel is simulated with two levels of treatments—one covariate and an interaction effect between the treatment and covariate using model (2). For simplicity, we denote by treatment group 1 patients receiving a specific treatment and by treatment group 0 the control group and the outcomes are whether the patients recover from a particular disease or not. Using the notations introduced above, $\tau_{1}$ and $\delta_{1}$ are set to 0.6 and −0.6, respectively, in Figure 1a and $\tau_{1}$ and $\delta_{1}$ are set to 0.6 and −0.4, respectively, in Figure 1b. $\tau_{2}$ and $\delta_{2}$ are set to 0 for both simulated examples. The log odds for both examples are computed and plotted against treatment groups for models (1) and (2). In Figure 1a, the log odds of the logistic regression model are similar between the treatment group and the control group. The effectiveness of the treatment group is not obvious for the logistic regression model. The log odds are more spread out using the AbRelaTEs model which is reasonable and can be interpreted in our setting. Higher log odds suggest a high probability of recovering from the disease for patients with similar attributes receiving the treatment. Lower log odds, as observed for the treatment group, show that patients with different attributes (different weight range, age group, etc.) have a lower probability of recovering from the disease. These observations indicate that the treatment group can be recommended for patients sharing similar attributes (similar weight range, age group, etc.) using the AbRelaTEs model since the AbRelaTEs model also considers the attributes of the patients as discussed above. Similarly, in panel (b), even though the log odds are generally higher in the treatment group using the logistic regression model, the log odds computed from the logistic regression model are underestimated/overestimated for some patients. In addition, the treatment group can be recommended for patients sharing similar attributes based on the log odds using the AbRelaTEs model.

The purpose of the two simulated examples is to show that the AbRelaTEs model provides enhanced interpretations and more significant results that the logistic regression may fail to capture. In addition, it is clear from the toy examples that the term $\delta_{j}$ is proposed to detect the relative treatment effects. Our model’s applicability and interpretability will be further discussed and presented with some real data examples in the numerical analysis section. In the subsequent section, we will present the theoretical guarantees of the estimation procedure in our model setting.

## 3. Estimation and Asymptotic Theory

In this section, we provide some additional discussions on the AbRelaTEs model for estimation purposes. Subsequently, we present the maximum likelihood estimation procedure and discuss the asymptotic properties in our model setup.

Different treatment effect representations can be applied to represent whether a patient is in the treatment or control group. For instance, consider the case of two treatment groups; the treatment group can be represented by 1 if the patient is in the treatment group and by −1 if the patient is in the control group. Alternatively, the treatment group can be represented by 1 if the patient is in the treatment group and otherwise 0. Since treatment effects are measured differently in the AbRelaTEs model, the control group’s constraint can be differently set for the absolute and relative treatment effects. However, using the same representation has an advantage. Here, we simply provide some discussions of the parameter $\delta_{j}$ and show our model’s versatility by different specifications of the treatment variables. In this paper, we only considered and focused on the parameter $\delta_{j}$ being the relative treatment effect. Additionally, regardless of the choice of representing the treatment groups, the interpretations are similarly made for each treatment group at the patient level as discussed in Section 2.2.

We denote $\tau ={\left(\tau_{1},\tau_{2},...,\tau_{g-1}\right)}^{\prime }$ and $\delta ={\left(\delta_{1},\delta_{2},...,\delta_{g-1}\right)}^{\prime }$ as (g−1)×1 vectors of the absolute and relative treatment coefficients and let $\theta ={\left(\mu ,\tau^{\prime },\beta^{\prime },\delta^{\prime }\right)}^{\prime }$ be a (2g+p−1)×1 parameter vectors. In this paper, we set the *g*th group as the control group. The theoretical guarantees can be established using the setting discussed in the previous section for the parameters $\tau_{j}$ and $\delta_{j}$. The log-likelihood function l(θ) using the model (2) is given by

(10) $$l\left(\theta \right)=\sum_{j=1}^{g}\sum_{i=1}^{n_{j}}\left\{Y_{ij}\left(\mu +\tau_{j}+X_{ij}^{\prime }\beta \right)\left(1+\delta_{j}\right)-\log\left\{1+\exp\left[\left(\mu +\tau_{j}+X_{ij}^{\prime }\beta \right)\left(1+\delta_{j}\right)\right]\right\}\right\}.$$

The maximum likelihood estimator $\hat{\theta }$ is obtained by optimizing the log-likelihood function:

(11) $$\hat{\theta }=\arg\max_{\theta }l\left(\theta \right).$$

For parameter estimation and theoretical purposes, we expressed the model (2) in a matrix form. We denote by $T_{ij}={\left(T_{i,1},T_{i,2}...,T_{i,(g-1)}\right)}^{\prime }$ as a (g−1)×1 vector containing the treatment group information of *i*-th patient, e.g., if the *i*-th patient is in treatment group 1, the vector is shown as ${\left(1,0,...,0\right)}^{\prime }$ and $\tau ={\left(\tau_{1},...,\tau_{g-1}\right)}^{\prime }$ is the corresponding coefficient vector. Similarly, we let $R_{ij}={\left(R_{i,1},R_{i,2}...,R_{i,(g-1)}\right)}^{\prime }$ be a (g−1)×1 vector containing the treatment group information for the relative term and $\delta ={\left(\delta_{1},...,\delta_{g-1}\right)}^{\prime }$ is the corresponding coefficient vector. We define $T_{ij}^{*}\left(\delta \right)={\left(T_{i,1}\left(1+R_{ij}^{\prime }\delta \right),T_{i,2}\left(1+R_{ij}^{\prime }\delta \right)...,T_{i,(g-1)}\left(1+R_{ij}^{\prime }\delta \right)\right)}^{\prime }$ and $X_{ij}^{*}\left(\delta \right)={\left(X_{i,1}\left(1+R_{ij}^{\prime }\delta \right),X_{i,2}\left(1+R_{ij}^{\prime }\delta \right),...,X_{i,p}\left(1+R_{ij}^{\prime }\delta \right)\right)}^{\prime }$. Additionally, we let $W_{ij}\left(\delta \right)={\left(1+R_{ij}^{\prime }\delta ,T_{ij}^{{*}^{\prime }}\left(\delta \right),X_{ij}^{{*}^{\prime }}\left(\delta \right)\right)}^{\prime }$ be a (g+p)×1 vector and $\beta^{*}={\left(\mu ,\tau^{\prime },\beta^{\prime }\right)}^{\prime }$ be the corresponding coefficient vector. Let $\theta_{0}={\left(\beta_{0}^{{*}^{\prime }},\delta_{0}^{\prime }\right)}^{\prime }={\left(\mu_{0},\tau_{0}^{\prime },\beta_{0}^{\prime },\delta_{0}^{\prime }\right)}^{\prime }$ be the true parameter vector and Θ be the parameter space of $\theta_{0}$. We let ϕ(u) be defined by ϕ(u)=exp(u)/(1+exp(u)) and $Z_{ij}={\left(W_{ij}^{\prime }\left(\delta_{0}\right),V_{ij}^{\prime }{\beta_{0}}^{*}R_{i,1},V_{ij}^{\prime }{\beta_{0}}^{*}R_{i,2},...,V_{ij}^{\prime }{\beta_{0}}^{*}R_{i,(g-1)}\right)}^{\prime }$ be a (2g+p−1)×1 vector where $V_{ij}={\left(1,T_{ij}^{\prime },X_{ij}^{\prime }\right)}^{\prime }$. To establish the asymptotic properties of the maximum likelihood estimator, we need the following assumptions.

(A1) Define C=(−1,1). $\theta_{0}$ is an interior point of an open set in the parameter space $\Theta \subseteq R^{g+p}\times C^{g-1}$.
(A2) For all *i* and l=1,2,...,p, $E|X_{il}{|}^{k}<\infty$ for k=1,2,3,4.
(A3) $E(W_{ij}\left(\delta_{0}\right)W_{ij}^{\prime }\left(\delta_{0}\right))$ and $E\{\phi \left(W_{ij}^{\prime }\left(\delta_{0}\right)\beta_{0}^{*}\right)\left[1-\phi \left(W_{ij}^{\prime }\left(\delta_{0}\right)\beta_{0}^{*}\right)\right]Z_{ij}Z_{ij}^{\prime }\}$ are positive definite matrices.

The assumptions (A1)–(A3) are commonly seen in the proofs of consistency and asymptotic normality of the maximum likelihood estimator. We adjusted the assumptions to fit our model setup.

**Theorem** **1.** *(Consistency) Under assumptions (A1)–(A3), as $n_{j}\to \infty$ and n→∞, we have $\hat{\theta }\to_{p}\theta_{0}$.*

**Theorem** **2.** *(Asymptotic Normality) Under assumptions (A1)–(A3), as $n_{j}\to \infty$ and n→∞, we have:*

$$\sqrt{n}\left(\hat{\theta }-\theta_{0}\right)\to_{D}N\left(0,{\left[I\left(\theta_{0}\right)\right]}^{-1}\right),$$

*where $I(\theta_{0})$ is the expected Fisher information at $\theta_{0}$ and the expression is given in the Appendix A.*

**Remark** **1.** *In addition, since the AbRelaTEs model is a generalization of the logistic regression, it preserves other desirable properties: it can be shown that the AbRelaTEs model is identifiable and belongs to a full-rank exponential family with the assumptions.*

## 4. Numerical Analysis

We will present the estimation procedure for the simulation and real data analyses in this section. Firstly, the first partial derivatives of the log-likelihood function in (10) with respect to parameters μ, τ, β and δ are given by

(12) $$\frac{\partial l(\theta )}{\partial \mu }=\sum_{j=1}^{g}\sum_{i=1}^{n_{j}}\left\{Y_{ij}\left(1+R_{ij}^{\prime }\delta \right)-\frac{\exp(W_{ij}^{\prime }\left(\delta \right)\beta^{*})}{1+\exp(W_{ij}^{\prime }\left(\delta \right)\beta^{*})}\left(1+R_{ij}^{\prime }\delta \right)\right\},$$

(13) $$\frac{\partial l(\theta )}{\partial \tau_{j}}=\sum_{i=1}^{n_{j}}\left\{Y_{ij}T_{i,j}\left(1+R_{ij}^{\prime }\delta \right)-\frac{\exp(W_{ij}^{\prime }\left(\delta \right)\beta^{*})}{1+\exp(W_{ij}^{\prime }\left(\delta \right)\beta^{*})}T_{i,j}\left(1+R_{ij}^{\prime }\delta \right)\right\},$$

(14) $$\frac{\partial l(\theta )}{\partial \beta_{k}}=\sum_{j=1}^{g}\sum_{i=1}^{n_{j}}\left\{Y_{ij}X_{ik}\left(1+R_{ij}^{\prime }\delta \right)-\frac{\exp(W_{ij}^{\prime }\left(\delta \right)\beta^{*})}{1+\exp(W_{ij}^{\prime }\left(\delta \right)\beta^{*})}X_{ik}\left(1+R_{ij}^{\prime }\delta \right)\right\},$$

(15) $$\frac{\partial l(\theta )}{\partial \delta_{j}}=\sum_{i=1}^{n_{j}}\left\{Y_{ij}V_{ij}^{\prime }\beta^{*}R_{i,j}-\frac{\exp(W_{ij}^{\prime }\left(\delta \right)\beta^{*})}{1+\exp(W_{ij}^{\prime }\left(\delta \right)\beta^{*})}V_{ij}^{\prime }\beta^{*}R_{i,j}\right\},$$

for j=1,2,...,g−1 and k=1,2,...,p. Based on the Equations (12) and (15), there are no closed form solutions for the MLE $\hat{\theta }$. We applied the Newton–Raphson method to obtain the estimates. At (t+1)-th iteration, the estimates ${\hat{\theta }}^{\left(t+1\right)}$ are computed using the following equation:

(16) $${\hat{\theta }}^{\left(t+1\right)}={\hat{\theta }}^{\left(t\right)}-H^{-1}\left({\hat{\theta }}^{\left(t\right)}\right)s\left({\hat{\theta }}^{\left(t\right)}\right),$$

where s(θ) is the score function in Equations (12)–(15) and H(θ) is the second derivatives of the log-likelihood function (10). The iterations using Equation (16) are performed until convergence is attained.

In some cases, the optimal values of the parameters δ might fall outside the interval (−1, 1) in the optimization procedure. To overcome the issue, we conduct a reparameterization as $\delta_{j}=\frac{e^{\eta_{j}}-1}{1+e^{\eta_{j}}}$ where $\delta_{j}$ is a monotone increasing function of $\eta_{j}$, and we solve $\eta_{j}$ in the optimization.

Furthermore, the estimation procedure above is highly dependent on the initial values of the parameters. If there are two treatment groups, we propose the following estimation procedure. We first split the parameter space of $\delta_{1}$, which ranges from −1 to 1, into equally-spaced smaller grids, and we estimate the coefficient parameters $\beta^{*}$ for each grid value of $\delta_{1}$. The coefficient parameters are then estimated using the Newton–Raphson method. At (t+1)-th iteration, the estimates ${\hat{\beta }}^{*(t+1)}$ are computed using the following equation:

(17) $${\hat{\beta }}^{*(t+1)}={\hat{\beta }}^{*(t)}-H^{-1}\left({\hat{\beta }}^{*(t)}\right)s\left({\hat{\beta }}^{*(t)}\right).$$

The iterations using Equation (17) are performed until convergence is attained. Subsequently, the log-likelihood (10) is evaluated at $\hat{\theta }={\left({\hat{\beta }}^{{*}^{\prime }},{\hat{\delta }}_{1}\right)}^{\prime }$. The values of $\delta_{1}$ and $\beta^{*}$, which maximize the log-likelihood function, are selected as the estimates for ${\hat{\delta }}_{1}$ and ${\hat{\beta }}^{*}$.

The proposed estimation procedure not only removes the need to choose an initial value for $\delta_{1}$ but also searches through a fair number of $\delta_{1}$ values and selects the solution which maximizes (10). This approach is similar to a grid-search approach that is widely adopted in the threshold or change-point regression literature. It is useful to search for the solution when there is no closed-form solution for the parameter with acceptable computational costs when performing the grid-search approach for one parameter—however, the computational costs for the grid search procedure increase as the number of treatments increases. Therefore, if the number of treatments is more than 2, we apply the estimation procedure as described in Equation (16).

In the next subsection, we will present some simulation examples to evaluate the AbRelaTEs model’s performance.

### Simulation

In this section, some simulation studies are conducted to assess the performance of the AbRelaTEs model. We considered a similar data structure as in our real data examples where there are two treatment groups (treatment and control)—each group having a similar number of patients/participants. We compared the performances of the AbRelaTEs model and logistic regression model in terms of their estimation and classification rates.

To compare the classification rates of the AbRelaTEs model and logistic regression model, we produced 1000 data simulated with n=1000 using different parameter values. Subsequently, the sensitivity and specificity for 1000 different simulations were computed for each model and the results are displayed using box plots. The first two covariates $x_{i1}$ and $x_{i2}$ are independently simulated from a normal distribution with a mean of 0 and a variance of 1. The third covariate $x_{i3}$ is simulated from a Bernoulli distribution. We also include the interaction term between the treatment effects and the first covariate $t_{i1}x_{i1}$. The coefficient parameters are simulated from a uniform distribution from −2.5 to 2.5 ($\beta_{j,0}∼$ Uniform(−2.5, 2.5) for j=1,2,3,4). The absolute and relative treatment effect parameters are simulated using $\tau_{1,0}∼$ Uniform(0, 2) and $\delta_{1,0}∼$ Uniform(−0.7, −0.3) with $\tau_{2,0}=-\tau_{1,0}$ and $\delta_{2,0}=-\delta_{1,0}$. In addition, we produced another simulation with $\delta_{1,0}∼$ Uniform (0.3, 0.7) and all other settings remain unchanged.

The simulation procedure is similar to the classical logistic regression model. Firstly, the success probability shown below is computed using the specified settings for the parameter values. For each patient/participant *i* in the treatment group, the success probability is:

$$\begin{array}{c} \pi_{ij}=\frac{\exp[\left(\mu_{0}+\tau_{j,0}+x_{ij}^{\prime }\beta_{0}\right)\left(1+\delta_{j,0}\right)]}{1+\exp[\left(\mu_{0}+\tau_{j,0}+x_{ij}^{\prime }\beta_{0}\right)\left(1+\delta_{j,0}\right)]}. \end{array}$$

The binary response variable is generated from Bernoulli experiments with success probability $\pi_{ij}$. Once the binary responses are generated for each patient/participant, the coefficients are estimated using the estimation procedure we described earlier in this section. The sensitivity and specificity for the 1000 data simulated from different parameter values are then computed for each model.

Subsequently, we present the simulation settings for estimation purposes. The number of variables considered in our model setup is p=4. The covariates $x_{ij}$ are independently simulated from a normal distribution with a mean of 0 and a variance of 1 ($x_{ij}∼N\left(0,1\right)$). The coefficients for the covariates are set to $\beta_{0}={\left(-0.5,0.5,-0.5,0.5\right)}^{\prime }$. We considered both absolute and relative treatment effects where the coefficients of the absolute and relative treatment effects are set to $\tau_{1,0}=-1,\;\tau_{2,0}=0$ and $\delta_{1,0}=-0.5,\;\delta_{2,0}=0$. Additionally, we also considered $\delta_{1,0}=-0.3,0.3,0.5$ as other parameter settings remain unchanged. The number of observations was set to n=300,500,700,1000. The simulation and estimation procedures were similarly performed as described above. In total, 1000 simulation runs were conducted for each of the settings. The averages of the estimated coefficients, standard deviations, standard errors and coverage probabilities were reported for both models. Similar quantities were computed and reported for the classical logistic regression.

We also tested our model performance by simulating data from the logistic regression model with $\tau_{1,0}=-1,\;\tau_{2,0}=0$ and $\beta_{0}={\left(-0.5,0.5,-0.5,0.5\right)}^{\prime }$ with all other settings remain unchanged. In addition, we also considered the case when the absolute treatment effect was not significant and the relative treatment effect was significant. We set $\tau_{1,0}=0$ as all other settings remain unchanged.

Furthermore, we presented simulation results to demonstrate the performance of the AbRelaTEs model when interaction effects exist. Two covariates and two interaction terms were considered with coefficients set to $\beta_{0}={\left(-0.5,0.5,-0.5,0.5\right)}^{\prime }$. The interaction terms considered are the interaction effects between the treatment effects and covariates, that are $t_{i1}x_{i1}$ and $t_{i1}x_{i2}$ using the notations introduced in Section 3. The interaction terms are included in the covariate matrix in model (2) by the design of the matrix. The absolute and relative treatment effects are similarly set to $\tau_{2,0}=-\tau_{1,0}$ and $\delta_{2,0}=-\delta_{1,0}$.

**Figure 2:**
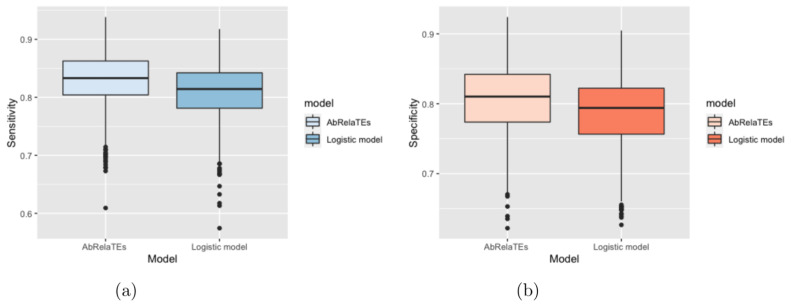
Box plots are displayed based on the sensitivity in panel (**a**) and specificity in panel (**b**) computed for the AbRelaTEs and logistic models over 1000 datasets simulated using different parameter values with $\delta_{1,0}$ simulated from a uniform distribution from −0.7 to −0.3.

**Figure 3:**
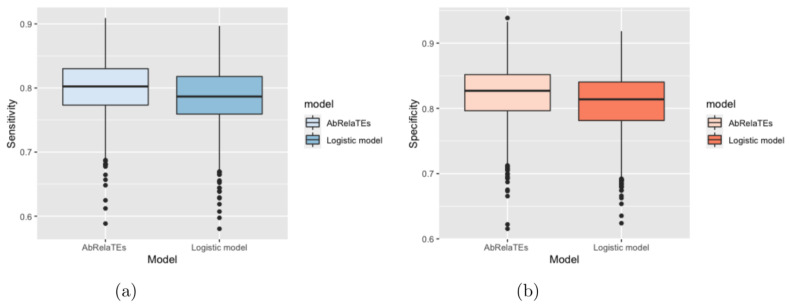
Box plots are displayed based on the sensitivity in panel (**a**) and specificity in panel (**b**) computed for the AbRelaTEs and logistic models over 1000 datasets simulated using different parameter values with $\delta_{1,0}$ simulated from a uniform distribution from 0.3 to 0.7.

Based on Figure 2 and Figure 3, the box plots show that the sensitivity and specificity are overall higher for the AbRelaTEs model based on the first quartiles, medians and third quartiles with similar variabilities between the AbRelaTEs and logistic regression models, suggesting that the AbRelaTEs model produces results with improved sensitivity and specificity when the relative treatment effects exist in the simulated datasets. The findings are reasonable since both models are based on the logistic regression model for binary classification which is the same type of classifier to achieve the optimal separation between two classes. Moreover, the relative treatment effects in the AbRelaTEs model helps improve the results for some data points in a certain range for continuous variables or of similar values for discrete variables (i.e., individualized effects), resulting in generally better sensitivity and specificity rates for the AbRelaTEs model, which were discussed in previous sections.

The results are shown in Table 1 for the case of $\tau_{1,0}=-1$, $\delta_{1,0}=-0.3$ whereas the results are given in the supplementary file for the cases of $\delta_{1,0}=-0.5,0.3,0.5$. The optimization is mainly based on the Newton–Raphson algorithm in model (17). The code can be obtained from the authors upon request or downloaded from Github. Based on the results in Table 1, the mean estimate for $\delta_{1}$ improves and approaches −0.3 as *n* increases from 300 to 1000. It was also observed that the standard deviation and standard error for the relative effect term decreases as the sample size increases. Similarly, the average estimate, standard deviation, and standard error improve for $\tau_{1}$ as *n* increases. For other coefficients, the average estimates are already closed to the specified coefficients $\beta_{0}={\left(-0.5,0.5,-0.5,0.5\right)}^{\prime }$ when n=500 whereas the standard deviations and standard errors improve as the sample size increases. On the other hand, the estimates for the coefficients using the logistic regression model are similar for all sample sizes. One interesting finding is that the coverage probability for the absolute effect term $\tau_{1}$ decreases from 0.690 to 0.279 for the logistic regression model as the sample size increases. This significant observation suggests that the logistic regression model might fail to capture or explain the absolute treatment effect when the relative treatment effect is significant as the sample size increases. We will further explore this aspect in the real data examples. Similar findings were also observed for the cases $\delta_{1,0}=-0.5,0.3,0.5$.

In addition, Table 2 shows that the AbRelaTEs model performance is comparable to that of the logistic regression model when $\delta_{1,0}=0$ (i.e., no relative treatment effects). It was observed that the average estimate for $\delta_{1}$ significantly improves as the sample size increases with improved standard deviation and standard error. The coefficient estimates obtained from the AbRelaTEs model were seen to be comparable to the logistic regression model even when n=300. The standard deviations and standard errors improve as the sample size increases. Similar findings are observed for the case of $\tau_{1,0}=0$ (i.e., no absolute treatment effects) and $\delta_{1,0}=-0.5,-0.3,0$, which are shown in the supplementary file. The coverage probability of the treatment effect using the logistic regression decreases as the magnitude of the relative treatment effect increases, which suggests that the logistic regression model might fail to capture any treatment effects if the relative treatment effect is significant. These findings suggest that the AbRelaTEs model can also model datasets when the relative treatment effect is not significant. This will also further be shown and discussed using the MEPARI-2 dataset in the real data analysis part.

The performance of the AbRelaTEs model is desirable when interaction effects exist as shown in Table 3. On the other hand, the estimates of the coefficients for the treatment effects, covariates, and interaction effects are similar for varying sample sizes. The coverage probabilities for the treatment effect in the logistic regression model are also similar which are approximately 94% for different sample sizes and the coefficient estimates for the treatment effect are similar to the coefficient estimates for the absolute treatment effect in the AbRelaTEs model. However, as the sample size increases, the coverage probabilities for the covariates and interaction terms substantially decrease from 66% to approximately 20%. In Table 4, the AbRelaTEs model outperforms the logistic regression model when interaction effects exist with the relative treatment effect being 0.5—as observed in Table 4. The coefficient estimates are similar for the treatment effects, covariates, and interaction effects for different sample sizes using the logistic regression model. The coverage probabilities for the parameters decrease as the sample size increases. The coverage probability decreases from approximately 82% to 40% as the sample size increases from 300 to 1000. These suggest that the logistic regression model is able to capture the absolute treatment effect but the performance is poor in capturing the covariates and interaction effects for a larger sample size when $\delta_{1,0}=-0.5$ and the logistic regression model is poor in capturing the absolute treatment effect when $\delta_{1,0}=0.5$. For a smaller magnitude of the relative treatment effects, the performance of the logistic regression is reasonable.

From these simulation examples, we showed that the AbRelaTEs model outperforms the logistic regression under no interaction/with interaction effect settings. We note that $\delta_{j}$ should not be interpreted as interaction effects as used in the classical logistic regression models based on our theoretical arguments and numerical results (i.e., it is truly a relative effect indicator). In addition, we also demonstrated that the AbRelaTEs model was able to estimate the parameters simulated by the logistic regression (i.e., no relative treatment effect). In addition, the estimates produced by the logistic regression model will result in incorrect log odds and odds ratio as the model is incapable of capturing the relative treatment effects, as shown in the simulation results. Consequently, decision making and developing an optimal treatment plan based on the log odds and odds ratio will be challenging. These simulation examples suggest that the AbRelaTEs model can be used as a new benchmark model, as mentioned in the previous section. In the subsequent section, we will show that the AbRelaTEs model is able to capture significant treatment effects through either the absolute or relative or both ways.

## 5. Real Data

We present the statistical analyses of four different datasets using our model and the classical logistic regression model. We aimed to show the flexibility and interpretability aspects of the AbRelaTEs model in handling different clinical trials datasets with detailed analyses. In addition, it is also important to note that the AbRelaTEs model is capable of capturing the treatment effects of a randomized controlled trial through either the relative or the absolute treatment effect terms, which we will show through four real data examples in the following subsections. Table 5 shows the three possible outcomes of whether a treatment effect is significant in the AbRelaTEs model.

### 5.1. Sepsis Data

This section will explore a randomized controlled trial on the use of synbiotics as a treatment for sepsis. The occurrence of sepsis is due to systemic inflammation and circulatory compromise by means of infection. Sepsis is a leading cause of death in infants with a 5–60% fatality rate [19]. Currently, there are no efficient ways to prevent sepsis. A dataset was obtained from a randomized controlled trial study conducted on 4556 rural Indian newborns [20]. The infants were randomized into the synbiotic group (2278) and placebo (2278). Among the 4556 infants, 4326 completed the study. Synbiotics are combinations of prebiotics and probiotics (Lactobacillus plantarum plus fructooligosaccharide) in the trial. The primary outcome of interest is the combination of sepsis and death.

The covariates that are significant in our analysis are birth weights (in grams) and sex. The weight variable is transformed using a reciprocal transformation. The estimation results based on the AbRelaTEs model and logistic regression model are shown in Table 6. The results show that the variables are all significant for the logistic regression model except for the variable birth weight. On the other hand, only the absolute treatment effect term is not significant in our model, while other covariates are significant. This illustrates that the relative treatment effect is significant for the data. Table 7 displays our model’s estimation results after removing the absolute treatment effect term. The results show that the relative treatment effect term and the covariates are significant. There is one difference in the coefficient sign of the relative treatment effect term we will address in the interpretation part.

The log-odds of infants having sepsis or death change by (−6.755)∗(1+0.240)=−8.376 (synbiotic) and −6.755 (control) for every unit increase in weight. The odds of having sepsis or death in infants are exp(0.225∗(1+0.240))=1.322 (synbiotic) and exp(0.225)=1.252 (control) higher for the male infants than the female infants. We now interpret the results by comparing them between treatment effects. After computing the odds ratios for each weight and gender, the odds ratios are consistently smaller than 1, which shows that the treatment is effective for all weight groups and both genders. The interpretation is also consistent with that of the logistic regression model, even though the treatment effect appears as a relative term in our model. Furthermore, after removing the absolute treatment effect, the positive coefficient sign of the relative effect term without the absolute treatment term implies that there is a multiplier effect on the log-odds uniformly for all infants receiving the treatment. Additionally, the sensitivity and specificity for the AbRelaTEs model are 60.5% and 50.7% while the sensitivity and specificity are 39.4% and 75.6% for the logistic regression model. Both interpretations and results for these data show that the AbRelaTEs model not only gives interpretations that are consistent with the logistic regression model but also shows that the birth weight variable is actually a significant predictor under our framework. On the other hand, the sensitivity of 39.4%, which is smaller than 50%, calculated from the logistic regression, is problematic as it leads to conclude that synbiotics are not effective and that the interpretation can be wrong.

### 5.2. MEPARI-2 Data

In this subsection, we will explore a randomized controlled trial on meditation or exercise for an acute respiratory infection prevention (MEPARI-2) dataset [21]. It is of interest to investigate whether interventions such as meditation and exercise help reduce acute respiratory infection (ARI) outcomes and whether self-reported psychosocial scores from the participants are associated with ARI outcomes. Out of 413 participants enrolled in the study, there were 389 data points after removing the participants with missing information and incomplete data during the study.

Based on the estimation results in Table 8, the exercise group was found to be significant and the meditation group was removed from the model since it was not significant. The results show that the relative treatment effect term was not significant in the AbRelaTEs model with a high *p*-value. In addition, the coefficient estimates for the treatment group, age, self-reported psychosocial scores, and interaction terms are closed to the estimates from the logistic regression model. This shows that the AbRelaTEs model produces results that are similar to the logistic regression model when the relative treatment effect term is not significant, and the absolute treatment effect is significant. We also note that the coefficients, standard errors, and *p*-values are the same after we remove the relative treatment effect term from our model. The sensitivity and specificity for both models are the same which are 56.8% and 59.5%, respectively. Moreover, since the interpretations under this scenario will be similar to the interpretations using the logistic regression model by interpreting each predictor’s effects, we will not discuss it further.

### 5.3. Influenza Data

In this subsection, we investigated a flu vaccination dataset [22]. Vaccination is essential in preventing the infection and transmission of influenza viruses. To investigate the effect of vaccinating children in the household environment, 796 households were enrolled in this study and randomized into the vaccination group (479 households) or control group (317 households) with at least one child. Since there are adults who are not vaccinated assigned to the treatment group and adults who are vaccinated in the control group, we focused on the effect of vaccination on children. The response variable of interest is whether the individual is infected or not.

The covariates that we found to be significant and include in our analysis are round (1,2,3) and the HAI titer level (0,1,2). The estimation results for our model and the logistic regression model are given in Table 9. The *p*-values are not significant for the treatment effect (in logistic regression) and round (in AbRelaTEs model). The results based on the AbRelaTEs model show the relative, absolute treatment effects and HAI titer level are significant. Since there are three rounds of sera collections in the study, we retained the variable as it indicates the period of time the data are collected though it is not significant.

For every increase in HAI titer level, the children’s log-odds have an influenza change by −0.59∗(1−0.242)=−0.447 for the vaccinated group and decreases by 0.59 for the control group. After computing the overall effects, it was found that the vaccinated treatment was beneficial for all HAI titer levels across different rounds. In addition, the sensitivity and specificity for the AbRelaTEs model are 62.5% and 64.6% while the sensitivity and specificity for the logistic regression model is 33.9% and 74.4%. Therefore, vaccination is highly recommended for all children based on the results. Again, a sensitivity of 33.9% calculated from the logistic regression may be meaningless.

### 5.4. COVID-19 Data

Our following statistical analysis was to explore a randomized controlled trial on the use of the hydroxychloroquine drug on the novel coronavirus disease (COVID-19). There have been many studies on the novel coronavirus disease 2019 (COVID-19) since its outbreak. To date, there are still many ongoing types of research with continued efforts to find effective antiviral treatments for patients with COVID-19. The dataset considered for our analysis was obtained from one of the studies on hydroxychloroquine [23]. The purpose of the study was to investigate whether hydroxychloroquine can prevent symptomatic infection after SARS-CoV-2 exposure. A total of 821 patients with occupational or household exposure to people with confirmed COVID-19 infection were enrolled in the study. The patients were randomized into hydroxychloroquine and placebo within four days of exposure. The primary outcome of the study was the incidence of laboratory-confirmed COVID-19 infections. The predictors considered for the analysis are treatments (hydroxychloroquine and placebo), age, and weight. Additionally, other independent variables include data on patients having symptoms (cough, shortness of breath, difficulty breathing, fever, chills, rigors, myalgia, headache, sore throat, new olfactory, taste disorders, and diarrhea). After removing patients with missing information, there were 746 patients for the statistical analysis. The number of patients for each variable in each treatment is presented in Table 10.

The estimation results using the classical logistic regression model and the AbRelaTEs model are presented in Table 11. In addition, the weight variable is transformed using a reciprocal transformation (weight${}^{*}$ = 1/(weight/500)) where weight${}^{*}$ is the transformed variable. The scaling factor is used here so that the magnitude of the estimated coefficient is not large. BMI is not available as the height data are not available. The results show that the absolute treatment effect is not significant using the classical logistic regression model and all predictors except age and number of symptoms are also not significant. These logistic regression-based results suggest that the hydroxychloroquine treatment is not significant in predicting the probability that a patient who has COVID-19 infection. They are consistent with other earlier and recent studies on the hydroxychloroquine drug [24,25,26] which show that the hydroxychloroquine treatment has no clinical benefits or does not prevent illness compatible with COVID-19 [23]. In contrast to our analysis, the aforementioned studies analyzed the data using statistical methods such as survival models, hazard/risk ratios and Fisher’s exact test which is not directly comparable in our case. However, compared with the fitted AbRelaTEs model, the resulting *p*-values associated with logistic regression in Table 11 are doubtful; they lack interpretability, which raises questions concerning whether the logistic regression model is correctly specified and has sufficient detecting power to detect the predictors’ effectiveness.

We will now interpret the results of our model shown in Table 11. The interpretations of the treatment effects can be made in two ways—between and within treatment groups. For within treatment groups, the effect of each covariate is illustrated and discussed using the odds. For a unit increase in age, the log-odds of people having COVID-19 change by −0.099∗(1−0.513)=−0.048 (hydroxychloroquine) and −0.099∗(1+0.513)=−0.150 (placebo). The log-odds changes are −0.947∗(1−0.513)=−0.461 (hydroxychloroquine) and −0.947∗(1+0.513)=−1.433 for a unit increase in weight${}^{*}$, respectively. Again, the significance of the age and weight is due to the effectiveness of hydroxychloroquine relative to placebo. Furthermore, the log-odds of people contracting COVID-19 increased by (0.833+0.463)∗(1−0.513)=0.631 (hydroxychloroquine) and (0.833−0.463)∗(1+0.513)=0.560 (placebo) for every additional number of symptoms. The odds presented for each treatment are not directly comparable between two treatments. The effects of between treatment effects are discussed later.

With regard to the between treatment group interpretations, all covariates are considered when making comparisons between the treatment groups. The interpretations are made using the overall effects for a group of patients in certain age and weight groups with or without symptoms (individualized effects). We first made interpretations for patients who did not show any symptoms. The odds ratios for patients were compared between the hydroxychloroquine and placebo groups to identify patients of the age group and weight range which would benefit from the treatment. For instance, the odds ratio for patients with no symptoms can be computed as follows:

(18) $$\frac{\mathrm{Odds}(\mathrm{hydroxychloroquine})}{\mathrm{Odds}(\mathrm{placebo})}=\frac{\exp\{\left(-3.279-0.099\mathrm{age}-0.947{\mathrm{weight}}^{*}\right)\left(1-0.513\right)\}}{\exp\{\left(3.279-0.099\mathrm{age}-0.947{\mathrm{weight}}^{*}\right)\left(1+0.513\right)\}},$$

where Odds(hydroxychloroquine) and Odds(placebo) are the odds of having COVID-19 for patients receiving the respective treatments. We note that the results are not evident and certain as to which treatment group consistently outperforms the other for all age and weight groups. It is also worth noting that the hydroxychloroquine treatment is only beneficial for certain age and weight groups which are our goal to identify here. The hydroxychloroquine treatment is more effective if the odds ratio is less than 1 and is less effective if the odds ratio is greater than 1. The odds ratios are shown in Table 12 for selective age, weight variable, and symptoms since the odds ratios of other age, weight, and symptom groups can be similarly computed. For instance, for patients who do not have any symptoms with the age of 30 and weight (pounds) between 139 and 385, the odds ratio is between 0.106 and 0.991. The odds ratio is between 0.114 and 0.921 for patients who have one symptom with the same age and weight between 145 and 385. We will first interpret the results for patients who do not show any symptoms. The odds of having COVID-19 are lower for patients receiving hydroxychloroquine treatment with ages ranging from 18 to 25 and weight above 122 pounds. The hydroxychloroquine treatment has a lower odds than the placebo in contracting the disease for patients who weigh more than 139 pounds and in the age group of 25–30 with no symptoms. Furthermore, patients who are in the age range of 30–40 and weigh at least 198 pounds have a lower odds ratio. Finally, for patients aged between 40 and 50 that weigh more than 335 pounds, the odds of contracting COVID-19 are lower for the hydroxychloroquine treatment group.

Subsequently, we will interpret the results for patients who show one symptom. Patients who are in the age group of 18–25 and weigh more than 105 pounds have lower odds of contracting COVID-19 in the hydroxychloroquine treatment. The odds of contracting the disease are lower for patients in the age range between 25 and 30 and those with weights above 145 pounds receiving hydroxychloroquine treatment. The odds are lower for the hydroxychloroquine treatment group within the age groups of 30–40 and 40–50, who are at least 202 pounds and 348 pounds in the respective age group. Similar interpretations can be made for patients who show up to ten symptoms (2, 3, ⋯, 10). It is also important to note that a more accurate weight range can be obtained for a given age so that the effects of the hydroxychloroquine treatment can be further explored. We consider a reasonable age range for easier interpretations as a group and identify the corresponding weight range where the hydroxychloroquine treatment is deemed beneficial.

**Figure 4:**
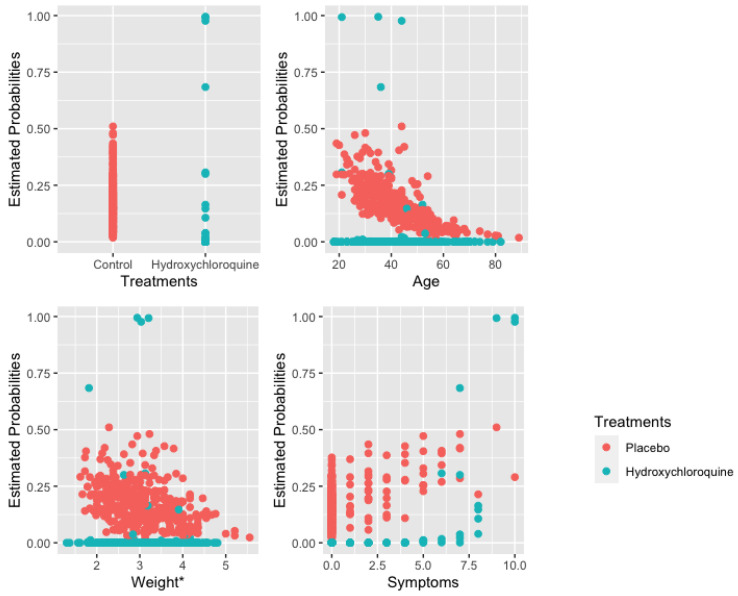
Estimated probabilities for each patient for hydroxychloroquine treatment and placebo.

Figure 4 illustrates the estimated probabilities of having COVID-19 computed using the estimated coefficients from the AbRelaTEs model against the covariates in the model (treatments, age, weight and number of symptoms) for each patient in the dataset. The comparisons and discussions made above based on the odds are similarly observed in Figure 4. The interpretations based on the odds of contracting the disease are similar to the estimated probabilities that a patient is infected. However, the figure provides additional insights. It is observed that there are two separate groups of patients undergoing hydroxychloroquine treatment based on the estimated probabilities. The separation is more apparent when looking at the plot for treatments, age, and weight. Further investigation shows that the group of patients with higher estimated probabilities experience all ten symptoms while another group of patients with lower estimated probabilities of contracting COVID-19 show fewer symptoms. These suggest the fact that the hydroxychloroquine treatment helps lower the probability of having COVID-19 with fewer symptoms. Furthermore, the sensitivity and specificity for the AbRelaTEs model are 78.9% and 86.2% while the sensitivity and specificity are 73.7% and 90.1% for the logistic regression model. Based on the significant results and interpretations, since the treatment is beneficial for a certain group of people but not for every patient, they should consult a medical doctor before taking the drug.

### 5.5. Discussion

The AbRelaTEs model not only produces significant treatment effects with better interpretability through the real data examples but the model can also be applied to other medical data in epidemiology. When using other medical data in epidemiology such as in the case-control or cohort studies, it is often of interest to model the exposure and the response by including other risk factors. The exposure in such studies can be captured by either the absolute or relative “exposure” effect terms in the AbRelaTEs model. If the absolute exposure effects are significant and relative exposure effects are not significant, the interpretations are similar to the logistic regression. On the other hand, if both terms are significant, the interpretations can be made based on “between” and “within” exposure effects together with the risk factors. Compared to other multivariable methods such as the logistic regression, the main advantage of the AbRelaTEs model is that it allows researchers to interpret results based on each exposure specific to each risk factor so that a subgroup of individuals with exposure and a specific risk factor can be identified as having lower/higher risk in relation to the response of interests.

Similar to the logistic regression, the odds ratio can be reported for the AbRelaTEs model. In addition, a more detailed odds ratio can be computed and tabulated as in Table 12 to report which subgroups of individuals/patients could benefit the most from or be least affected by the exposure/treatments.

With the four real data examples we presented, we summarize the essential findings of the treatment effects that we discussed in the previous subsections in Table 13 and include more details, e.g., covariates, response, treatment effects, to provide an overview of the results of the four real datasets for the AbRelaTEs and logistic regression models in Table 14. This shows that significant treatment effects are better explained in terms of absolute or relative or both ways with increased flexibility in the AbRelaTEs model. In addition, we also showed that the treatment effects can also be interpreted using individualized information for each patient/participant. In contrast, the widely used multivariable methods were not able to detect these features.

The synbiotic treatment was found to be beneficial for all infants with sepsis using the AbRelaTEs model. The birth weights and gender of infants were found to be significant variables in predicting sepsis. It was found that infants receiving the synbiotic treatment have lower odds of having sepsis as compared to the control group as weight increases. Furthermore, the odds were higher for male infants as compared to female infants for the synbiotic and control groups.

Acute respiratory infection can be improved by engaging in more physical activities (exercise group). It was found that the odds of having ARI decrease as age increases and the MASS score increases. On the other hand, the odds of having ARI increase as the SF12 score increases.

Additionally, the flu vaccination is recommended for children based on the AbRelaTEs model. A higher HAI titer level was also found to lower the odds of contracting a flu.

For the COVID-19 dataset, the hydroxychloroquine treatment, symptoms, age, and weight were found to be significant using the AbRelaTEs model. The odds of contracting COVID-19 decrease as the age and weight${}^{*}$ increase. Furthermore, a higher number of symptoms is related to increased odds of having COVID-19. The hydroxychloroquine treatment for COVID-19 was found to be beneficial for specific groups of patients with certain symptoms, age, and weight, resulting in the treatment being suitable as a precision medicine (see Table 12). Therefore, people should consult a medical doctor before taking the drug.

## 6. Conclusions

In this paper, a more general logistic regression was proposed to model randomized controlled trials, which allows us to compare different treatment effects absolutely and relatively due to the AbRelaTEs model’s flexibilities. Our model maintains the CIPS properties as mentioned in the introduction and is highly flexible in modeling randomized controlled trials’ data with absolute or relative or both effects. To identify the treatment effects, we observed the absolute and relative treatment effects. The absolute treatment effect $\tau_{j}$ is an overall treatment effect while the relative treatment effect $\delta_{j}$ is a treatment effect relative to the baseline control group. If $\tau_{j}\neq 0$, there is an absolute treatment effect. There is a relative treatment effect if $\delta_{j}\neq 0$. In both cases, the treatment groups are effective. In addition, the signs of the treatment effects are important. If we investigate whether a drug is effective in curing a disease, then significant absolute treatment effect with a positive sign implies that the drug is effective. On the other hand, if we investigate whether a treatment is effective in lowering the likelihood of being infected by a disease, a significant absolute treatment effect with negative sign signifies that the treatment is effective. In both cases, $\delta_{j}$ can be positive or negative as the effectiveness of the treatment for patients depends on the patients’ attributes which are the individualized effects. Furthermore, the epidemiologists can compute a score based on $\left(\mu +\tau_{j}+X_{ij}^{\prime }\beta \right)\left(1+\delta_{j}\right)$. We can use a score of 0 as a benchmark, i.e., a probability threshold of 0.5. If $\left(\mu +\tau_{j}+X_{ij}^{\prime }\beta \right)\left(1+\delta_{j}\right)>0$, then the treatment groups are viewed as effective. If $\left(\mu +\tau_{j}+X_{ij}^{\prime }\beta \right)\left(1+\delta_{j}\right)<0$, then the treatment groups are viewed as ineffective. If the probability threshold is taken to a different value other than 0.5, the cut-off value 0 should also be changed accordingly.

Furthermore, the AbRelaTEs model can be interpreted in two ways—“between” and “within” treatment effects. When interpreting the “within” treatment effects, each individual predictor’s effects can be interpreted. Additionally, the “between” treatment effects allow us to make interpretations using the information of all covariates from each patient/participant in the data. The overall effects of a patient or a certain group of people sharing the same attributes known as the individualized effects are then compared between treatments. This enables us to make recommendations if a treatment is suitable for the general public or a specific group of people, allowing us to determine whether or not a treatment can be treated as a precision medicine.

In addition, the AbRelaTEs model has several advantages if we consider using a logistic regression model with treatment-specific coefficients $\beta_{j}$ for $X_{ij}$ given in model (19):

(19) $$\mathrm{logit}\left(\pi_{ij}\right)=\mu +\tau_{j}^{*}+X_{ij}^{\prime }\beta_{j}$$

for $i=1,2,...,n_{j}$ and j=1,2,...,g.

There will be three additional difficulties for such a general framework (19): (1) $\tau_{j}^{*}$ may not be significant due to treatment-specific coefficients for $X_{ij}$; (2) for medical data (i.e., clinical trials), $X_{ij}^{\prime }$s are often measured at the baseline, and $X_{ij}^{\prime }\beta$ are used as baseline characteristics in order to test whether the treatment indicator $\tau_{j}^{*}$ is significant or not. In a logistic regression model with treatment-specific coefficients, $\beta_{j}$s can be very different, and the interpretations of μ and $\tau_{j}^{*}$ can be difficult; and (3) the estimation of $\beta_{j}$s can be difficult. In addition, it is not feasible to define an overall relative effect for the treatment *j*. In contrast, in the AbRelaTEs model, we only need to estimate the relative treatment effect $\delta_{j}$, and all interpretations presented in this paper are valid. Furthermore, the AbRelaTEs model can be viewed as a bridge between the classical logistic regression model for medical data and the logistic regression model with treatment-specific coefficients for each predictor.

Similarly, the interpretations on the “between” and “within” group effects can be made when analyzing medical data in epidemiological studies (e.g., case-control studies or cohort studies) using the AbRelaTEs model. The groups of individuals or people with different exposure status or degree of exposure in epidemiology are used to study the absolute and relative group effects in the AbRelaTEs model. The main advantage of the AbRelaTEs model in analyzing such data is to better interpret the effects of the exposure levels on the response variable specific to each category in the risk factors, which is known as the “individualized” effect as discussed in the previous sections.

In addition, we showed that our model is capable of modeling the absolute and relative treatment effects through simulation examples. Moreover, it was also shown through four real-world randomized controlled trials data that our model is highly interpretable, resulting in better understandings of the treatment effects. In addition, it is also established that the model preserves desired theoretical properties such as consistency and asymptotic normality under regularity conditions. These properties suggest that the AbRelaTEs model can be used as a new benchmark model for modeling randomized controlled trials. The AbRelaTEs model which considers the treatment effects can be further extended to accommodate two-way effects.

Finally, our model can be extended to response variables being continuous or semi-continuous, and predictors being high dimensional. We can also specify the relative effect indicators $\delta_{j}$ to be functions of predictors. We will consider these topics in the future research.

## Figures and Tables

**Table 1:** Estimate, standard deviation (SD), standard error (SE), and coverage probability (CP) when $\tau_{1,0}=-1$ and $\delta_{1,0}=-0.3$ with 1000 simulation runs for the AbRelaTEs model and logistic regression.

		AbRelaTEs Model				Logistic Regression Model			
*n*	Parameter	Estimate	SD	SE	CP	Estimate	SD	SE	CP
300	$\delta_{1}$	−0.266	0.272	0.274	0.937	-	-	-	-
	$\tau_{1}$	−1.100	0.517	0.501	0.933	−0.737	0.195	0.190	0.690
	$\beta_{1}$	−0.514	0.178	0.173	0.949	−0.430	0.132	0.134	0.920
	$\beta_{2}$	0.532	0.179	0.176	0.945	0.444	0.134	0.133	0.920
	$\beta_{3}$	−0.509	0.171	0.173	0.957	−0.428	0.134	0.135	0.909
	$\beta_{4}$	0.516	0.182	0.174	0.944	0.432	0.140	0.136	0.908
500	$\delta_{1}$	−0.274	0.215	0.206	0.945	-	-	-	-
	$\tau_{1}$	−1.079	0.381	0.359	0.941	−0.752	0.152	0.146	0.588
	$\beta_{1}$	−0.509	0.136	0.131	0.949	−0.429	0.104	0.102	0.885
	$\beta_{2}$	0.504	0.134	0.133	0.944	0.425	0.104	0.103	0.876
	$\beta_{3}$	−0.508	0.141	0.132	0.935	−0.427	0.108	0.104	0.879
	$\beta_{4}$	0.509	0.134	0.133	0.949	0.429	0.103	0.102	0.893
700	$\delta_{1}$	-0.273	0.183	0.172	0.942	-	-	-	-
	$\tau_{1}$	−1.023	0.292	0.283	0.941	−0.731	0.126	0.123	0.418
	$\beta_{1}$	−0.502	0.115	0.111	0.936	−0.426	0.090	0.088	0.840
	$\beta_{2}$	0.502	0.108	0.110	0.952	0.427	0.083	0.086	0.875
	$\beta_{3}$	−0.506	0.121	0.113	0.938	−0.430	0.094	0.087	0.848
	$\beta_{4}$	0.509	0.115	0.112	0.948	0.431	0.092	0.087	0.857
1000	$\delta_{1}$	−0.297	0.144	0.139	0.943	-	-	-	-
	$\tau_{1}$	−1.039	0.248	0.239	0.947	−0.732	0.108	0.102	0.279
	$\beta_{1}$	−0.508	0.094	0.092	0.951	−0.427	0.073	0.072	0.824
	$\beta_{2}$	0.506	0.095	0.093	0.945	0.425	0.073	0.073	0.823
	$\beta_{3}$	−0.505	0.092	0.094	0.957	−0.424	0.071	0.071	0.802
	$\beta_{4}$	0.510	0.091	0.093	0.954	0.427	0.071	0.070	0.828

**Table 2:** Estimate, standard deviation (SD), standard error (SE), and coverage probability (CP) when $\tau_{1,0}=-1$ and $\delta_{1,0}=0$ with 1000 simulation runs for the AbRelaTEs model and logistic regression.

		AbRelaTEs Model				Logistic Regression Model			
*n*	Parameter	Estimate	SD	SE	CP	Estimate	SD	SE	CP
300	$\delta_{1}$	0.035	0.302	0.338	0.928	-	-	-	-
	$\tau_{1}$	−1.063	0.367	0.370	0.959	−1.013	0.211	0.203	0.946
	$\beta_{1}$	−0.515	0.164	0.163	0.950	−0.507	0.138	0.141	0.955
	$\beta_{2}$	0.531	0.164	0.165	0.950	0.524	0.141	0.142	0.958
	$\beta_{3}$	−0.512	0.157	0.162	0.964	−0.506	0.140	0.141	0.960
	$\beta_{4}$	0.519	0.168	0.164	0.946	0.512	0.147	0.142	0.954
500	$\delta_{1}$	0.034	0.253	0.258	0.944	-	-	-	-
	$\tau_{1}$	−1.048	0.275	0.275	0.960	−1.026	0.159	0.157	0.948
	$\beta_{1}$	−0.510	0.127	0.122	0.948	−0.508	0.111	0.107	0.953
	$\beta_{2}$	0.506	0.124	0.121	0.949	0.504	0.107	0.109	0.952
	$\beta_{3}$	−0.509	0.129	0.120	0.946	−0.505	0.111	0.108	0.951
	$\beta_{4}$	0.509	0.125	0.123	0.944	0.507	0.109	0.108	0.947
700	$\delta_{1}$	0.028	0.211	0.216	0.959	-	-	-	-
	$\tau_{1}$	−1.017	0.228	0.225	0.943	−1.005	0.129	0.131	0.951
	$\beta_{1}$	−0.504	0.108	0.103	0.947	−0.504	0.095	0.092	0.944
	$\beta_{2}$	0.503	0.100	0.101	0.959	0.503	0.087	0.090	0.965
	$\beta_{3}$	−0.506	0.112	0.107	0.945	−0.505	0.097	0.091	0.934
	$\beta_{4}$	0.509	0.106	0.104	0.945	0.509	0.094	0.092	0.948
1000	$\delta_{1}$	0.007	0.177	0.176	0.943	-	-	-	-
	$\tau_{1}$	−1.026	0.192	0.189	0.951	−1.006	0.109	0.110	0.955
	$\beta_{1}$	−0.509	0.089	0.086	0.949	−0.505	0.077	0.075	0.946
	$\beta_{2}$	0.506	0.088	0.087	0.951	0.502	0.075	0.073	0.951
	$\beta_{3}$	−0.505	0.087	0.089	0.952	−0.502	0.076	0.077	0.962
	$\beta_{4}$	0.509	0.085	0.081	0.952	0.505	0.074	0.078	0.965

**Table 3:** Estimate, standard deviation (SD), standard error (SE), and coverage probability (CP) when $\tau_{1,0}=-1$ and $\delta_{1,0}=-0.5$ with 1000 simulation runs for the AbRelaTEs model and logistic regression with two covariates and two interaction terms.

		AbRelaTEs Model				Logistic Regression Model			
*n*	Parameter	Estimate	SD	SE	CP	Estimate	SD	SE	CP
300	$\delta_{1}$	−0.512	0.145	0.147	0.956	-	-	-	-
	$\tau_{1}$	−1.030	0.145	0.143	0.950	−0.995	0.140	0.137	0.949
	$\beta_{1}$	−0.594	0.397	0.377	0.958	−0.295	0.147	0.135	0.669
	$\beta_{2}$	0.587	0.365	0.366	0.958	0.295	0.145	0.137	0.658
	$\beta_{3}$	−0.595	0.388	0.377	0.957	−0.296	0.141	0.139	0.670
	$\beta_{4}$	0.588	0.363	0.366	0.962	0.295	0.138	0.139	0.681
500	$\delta_{1}$	−0.512	0.130	0.127	0.947	-	-	-	-
	$\tau_{1}$	−1.020	0.124	0.123	0.952	−0.987	0.121	0.118	0.939
	$\beta_{1}$	−0.575	0.291	0.281	0.958	−0.297	0.122	0.120	0.587
	$\beta_{2}$	0.569	0.310	0.283	0.950	0.296	0.124	0.121	0.580
	$\beta_{3}$	−0.573	0.290	0.281	0.954	−0.295	0.123	0.123	0.552
	$\beta_{4}$	0.569	0.311	0.283	0.952	0.295	0.129	0.120	0.573
700	$\delta_{1}$	−0.498	0.089	0.089	0.953	-	-	-	-
	$\tau_{1}$	−1.011	0.084	0.086	0.954	−0.985	0.084	0.083	0.943
	$\beta_{1}$	−0.522	0.161	0.156	0.950	−0.292	0.088	0.082	0.304
	$\beta_{2}$	0.526	0.159	0.155	0.955	0.295	0.083	0.081	0.305
	$\beta_{3}$	−0.524	0.157	0.158	0.953	−0.295	0.084	0.085	0.331
	$\beta_{4}$	0.527	0.161	0.155	0.951	0.296	0.087	0.084	0.332
1000	$\delta_{1}$	−0.506	0.076	0.080	0.959	-	-	-	-
	$\tau_{1}$	−1.004	0.077	0.076	0.956	−0.977	0.075	0.074	0.938
	$\beta_{1}$	−0.523	0.142	0.140	0.957	−0.291	0.076	0.074	0.210
	$\beta_{2}$	0.528	0.136	0.141	0.956	0.295	0.073	0.072	0.205
	$\beta_{3}$	−0.523	0.138	0.142	0.959	−0.292	0.073	0.071	0.194
	$\beta_{4}$	0.525	0.137	0.141	0.960	0.293	0.075	0.073	0.208

**Table 4:** Estimate, standard deviation (SD), standard error (SE), and coverage probability (CP) when $\tau_{1,0}=-1$ and $\delta_{1,0}=0.5$ with 1000 simulation runs for the AbRelaTEs model and logistic regression with two covariates and two interaction terms.

		AbRelaTEs Model				Logistic Regression Model			
*n*	Parameter	Estimate	SD	SE	CP	Estimate	SD	SE	CP
300	$\delta_{1}$	0.503	0.147	0.147	0.956	-	-	-	-
	$\tau_{1}$	−1.043	0.178	0.173	0.948	−0.852	0.139	0.142	0.818
	$\beta_{1}$	−0.530	0.235	0.233	0.956	−0.622	0.159	0.159	0.905
	$\beta_{2}$	0.519	0.225	0.235	0.959	0.607	0.154	0.158	0.926
	$\beta_{3}$	−0.523	0.236	0.231	0.951	−0.619	0.155	0.159	0.905
	$\beta_{4}$	0.518	0.237	0.232	0.943	0.612	0.156	0.158	0.902
500	$\delta_{1}$	0.505	0.133	0.127	0.936	-	-	-	-
	$\tau_{1}$	−1.030	0.146	0.148	0.955	−0.843	0.120	0.122	0.741
	$\beta_{1}$	−0.525	0.193	0.199	0.957	−0.622	0.128	0.137	0.898
	$\beta_{2}$	0.518	0.197	0.195	0.953	0.615	0.137	0.135	0.883
	$\beta_{3}$	−0.525	0.193	0.194	0.952	−0.622	0.133	0.136	0.905
	$\beta_{4}$	0.523	0.201	0.197	0.953	0.619	0.139	0.137	0.868
700	$\delta_{1}$	0.506	0.089	0.089	0.954	-	-	-	-
	$\tau_{1}$	−1.015	0.102	0.103	0.951	−0.836	0.083	0.085	0.518
	$\beta_{1}$	−0.503	0.134	0.130	0.952	−0.605	0.094	0.094	0.820
	$\beta_{2}$	0.511	0.127	0.128	0.957	0.612	0.092	0.095	0.807
	$\beta_{3}$	−0.513	0.131	0.127	0.950	−0.610	0.093	0.095	0.804
	$\beta_{4}$	0.513	0.127	0.129	0.950	0.613	0.092	0.096	0.812
1000	$\delta_{1}$	0.501	0.079	0.080	0.955	-	-	-	-
	$\tau_{1}$	−1.004	0.091	0.091	0.957	−0.830	0.075	0.076	0.394
	$\beta_{1}$	−0.500	0.117	0.112	0.947	−0.601	0.084	0.084	0.804
	$\beta_{2}$	0.506	0.118	0.113	0.956	0.606	0.085	0.083	0.771
	$\beta_{3}$	−0.503	0.110	0.112	0.953	−0.603	0.079	0.082	0.800
	$\beta_{4}$	0.504	0.113	0.113	0.955	0.604	0.082	0.084	0.799

**Table 5:** Possible combinations if a treatment effect is significant using the AbRelaTEs model.

Absolute Treatment Effect	Relative Treatment Effect
Significant	Non significant
Non significant	Significant
Significant	Significant

**Table 6:** Estimation results from the AbRelaTEs model and the classical logistic regression for sepsis data.

Variables	AbRelaTEs Model			Logistic Regression Model		
	**Coefficient**	**Standard Error**	***p*** **-Value**	**Coefficient**	**Standard Error**	***p*** **-Value**
Relative Effect	−0.827	0.253	0.001	-	-	-
Intercept	-	-	-	−2.545	0.510	<0.0001
Absolute Effect	−14.257	24.235	0.556	−0.553	0.122	<0.0001
Birth Weight	−7.034	0.322	<0.0001	−0.036	1.359	0.979
Sex	0.406	0.147	0.0005	0.429	0.122	0.0004

**Table 7:** Estimation results from the AbRelaTEs model after removing the absolute treatment effect.

Variables	AbRelaTEs Model		
	**Coefficient**	**Standard Error**	***p*** **-Value**
Relative Effect	0.240	0.059	<0.0001
Birth Weight	−6.755	0.274	<0.0001
Sex	0.225	0.105	0.033

**Table 8:** Estimation results from the AbRelaTEs model and the classical logistic regression using the MEPARI2 dataset.

Variables	AbRelaTEs Model			Logistic Regression Model		
	**Coefficient**	**Standard Error**	***p*** **-Value**	**Coefficient**	**Standard Error**	***p*** **-Value**
Relative Effect	0.011	0.509	0.983	-	-	-
Intercept	-	-	-	-	-	-
Absolute Effect	−1.263	0.596	0.034	−1.262	0.594	0.034
Age	−0.019	0.011	0.068	−0.019	0.009	0.034
MASS	−0.036	0.225	0.872	−0.033	0.143	0.819
SF12	0.228	0.174	0.190	0.225	0.097	0.021
MASS × Exercise	0.305	0.139	0.028	0.306	0.133	0.021

Note: MASS × Exercise is the interaction term between massand exercise group.

**Table 9:** Estimation results from the AbRelaTEs model and the classical logistic regression for the flu vaccination data.

Variables	AbRelaTEs Model			Logistic Regression Model		
	**Coefficient**	**Standard Error**	***p*** **-Value**	**Coefficient**	**Standard Error**	***p*** **-Value**
Relative Effect	−0.242	0.117	0.039	-	-	-
Intercept	-	-	-	−1.661	0.379	<0.0001
Absolute Effect	−1.596	0.435	0.0002	−0.516	0.319	0.106
Round	−0.280	0.192	0.144	−0.272	0.164	0.097
HAI titer level	−0.590	0.256	0.021	−0.534	0.204	0.009

**Table 10:** Number of patients with symptoms/outcomes in different treatments.

Variables	Hydroxychloroquine (n=378)	Placebo (n=368)
Laboratory-confirmed diagnosis	10	9
Patients with symptoms	55	58

**Table 11:** Estimation results from the AbRelaTEs model and the classical logistic regression for COVID-19 data.

Variables	AbRelaTEs Model			Logistic Regression Model		
	**Coefficient**	**Standard Error**	***p*** **-Value**	**Coefficient**	**Standard Error**	***p*** **-Value**
Relative Effect	−0.513	0.149	0.0005	-	-	-
Intercept	-	-	-	−2.353	1.610	0.144
Absolute Effect	−3.279	1.462	0.024	0.406	0.397	0.306
Age	−0.099	0.025	<0.001	−0.059	0.027	0.030
Weight${}^{*}$	−0.947	0.369	0.010	−0.033	0.390	0.932
No. of symptoms	0.833	0.213	<0.001	0.552	0.086	<0.001
Symptoms × Treatment	0.463	0.204	0.022	0.116	0.082	0.158

Note: Symptoms × Treatment is the interaction term between the COVID-19 symptoms and treatments. Weight${}^{*}$ is the transformed weight variable.

**Table 12:** Computed odds ratio between the hydroxychloroquine treatment and placebo based on different covariate information. The weight variable is shown based on the original scale.

Age	Weight (Pounds)	No. of Symptoms	Odds Ratio
18	103–385	0	0.031–0.991
20	108–385	0	0.038–0.977
25	122–385	0	0.064–0.970
30	139–385	0	0.106–0.991
35	163–385	0	0.177–0.986
40	198–385	0	0.294–0.968
45	246–385	0	0.489–0.998
50	335–385	0	0.814–0.983
18	105–385	1	0.034–0.973
20	110–385	1	0.041–0.966
25	124–385	1	0.069–0.977
30	145–385	1	0.114–0.921
35	167–385	1	0.190–0.986
40	202–385	1	0.316–0.990
45	255–385	1	0.525–0.992
50	348–385	1	0.874–0.999

**Table 13:** Summary outcomes of the treatment effects using COVID-19, influenza, sepsis, and MEPARI-2 datasets.

Dataset	Absolute Treatment Effect	Relative Treatment Effect
MEPARI-2	Significant	Non significant
Sepsis	Non significant	Significant
COVID-19 and influenza	Significant	Significant

**Table 14:** Summary outcomes of the treatment effects and covariates using COVID-19, influenza, sepsis, and MEPARI-2 datasets for the AbRelaTEs model and logistic regression model.

Dataset	Response	Variables	AbRelaTEs	Logistic Regression
MEPARI-2	Acute respiratory infection	Relative treatment	Non significant ${}^{*}$	-
		Absolute treatment	Significant	Significant
		Age	Significant	Significant
		MASS	Non significant	Non significant
		SF-12	Significant ${}^{*}$	Significant
		MASS × Exercise	Significant	Significant
Influenza	Influenza among children	Relative treatment	Significant	-
		Absolute treatment	Significant	Non significant
		Round	Non significant	Non significant
		HAI titer level	Significant	Significant
Sepsis	Deaths or sepsis in infants	Relative treatment	Significant	-
		Absolute treatment	-	Significant
		Birth weight	Significant	Non significant
		Sex	Significant	Significant
COVID-19	COVID-19 infections	Relative Treatment	Significant	-
		Absolute treatment	Significant	Non significant
		Age	Significant	Significant
		Weight	Significant	Non significant
		No. of symptoms	Significant	Significant
		Symptoms × Treatment	Significant	Non significant

Note: An aster in the table means the model is reduced to the classical logistic regression. The relative treatment effect is not significant in the AbRelaTEs model for the MEPARI-2 dataset. The data are fitted using the AbRelaTEs model without the relative treatment effect. The results are the same as using the logistic regression model.

## Data Availability

The data used for the statistical analysis can be found in the research articles cited in the real data section.

## Appendix A

### Appendix A.1. Proofs of Theorem 1

We let the notation $U_{ij}$ denote $Y_{ij}|X_{ij}$ and $f(U_{ij}|\theta )$ be the likelihood function for each i,j given below:

(A1) $$f\left(U_{ij}|\theta \right)={\left(\frac{\exp[W_{ij}^{\prime }\left(\delta \right)\beta^{*}]}{1+\exp[W_{ij}^{\prime }\left(\delta \right)\beta^{*}]}\right)}^{Y_{ij}}{\left(\frac{1}{1+\exp[W_{ij}^{\prime }\left(\delta \right)\beta^{*}]}\right)}^{1-Y_{ij}}.$$

Taking log of the likelihood function (A1) gives us:

(A2) $$\log f\left(U_{ij}|\theta \right)=\left\{Y_{ij}W_{ij}^{\prime }\left(\delta \right)\beta^{*}-\log\left\{1+\exp\left[W_{ij}^{\prime }\left(\delta \right)\beta^{*}\right]\right\}\right\}.$$

From the main text, the log-likelihood function can then be rewritten using (A2) as

$$\begin{array}{c} l\left(\theta \right)=\sum_{j=1}^{g}\sum_{i=1}^{n_{j}}\log f\left(U_{ij}|\theta \right). \end{array}$$

Before we show the consistency results, we let $Q_{n}\left(\theta \right)$ be defined as

(A3) $$Q_{n}\left(\theta \right)=\frac{1}{n}\sum_{j=1}^{g}\sum_{i=1}^{n_{j}}\log f\left(U_{ij}|\theta \right).$$

Note that dividing the log-likelihood by *n* does not change the optimization in the main text but this allows us to easily obtain the consistency results. By assumptions (A2) and (A3), parameter identification is satisfied. Using the notations defined in the main text:

$$\begin{array}{cc} \left|\log\phi (W_{ij}^{\prime }\left(\delta \right)\beta^{*})|\right\} & =|\log\phi \left(0\right)+\lambda \left(W_{ij}^{\prime }\left(\tilde{\delta }\right){\tilde{\beta }}^{*}\right)W_{ij}^{\prime }\left(\delta \right)\beta^{*}|\\ \; & \leq \left|\log\phi \left(0\right)\right|+\lambda \left(W_{ij}^{\prime }\left(\tilde{\delta }\right){\tilde{\beta }}^{*}\right)\left|W_{ij}^{\prime }\left(\delta \right)\beta^{*}\right|\\ \; & \leq \left|\log2|+\{1+C\right|W_{ij}^{\prime }\left(\tilde{\delta }\right){\tilde{\beta }}^{*}\left|\}\right|W_{ij}^{\prime }\left(\delta \right)\beta^{*}|\\ \; & \leq \left|\log2|+\{1+C|\right|W_{ij}{\left(\delta \right)||}_{2}\left|\right|\beta^{*}{\left|\right|}_{2}\}||W_{ij}{\left(\delta \right)||}_{2}\left|\right|\beta^{*}{\left|\right|}_{2} \end{array}$$

where $\phi \left(u\right)=e^{u}/\left(1+e^{u}\right)$, $\lambda \left(u\right)=\phi^{\prime }\left(u\right)/\phi \left(u\right)$ and $\phi^{\prime }\left(u\right)$ is the first derivative with respect to *u*. The first equality is by mean value theorem. The second inequality is by triangular inequality. Third inequality is by continuity of λ(u) and last inequality is by Cauchy–Schwartz. By assumptions (A1) and (A2), the expectation of the moments and parameters are bounded. Similarly, $\log\{1-\phi \left(W_{ij}^{\prime }\left(\delta \right)\beta^{*}\right)\}$ is also bounded and $Y_{ij}$ is bounded too. By Lemma 2.2 in Newey and McFadden [27], $Q_{0}\left(\theta \right)$ has a unique maximum at $\theta_{0}$.

By weak law of the large number, we have point-wise convergence:

$$Q_{n}\left(\theta \right)=\frac{1}{n}\sum_{j=1}^{g}\sum_{i=1}^{n_{j}}\log f\left(U_{ij}|\theta \right)\to_{p}E\left(\log f\left(U_{ij}|\theta \right)\right)=Q_{o}\left(\theta \right).$$

Additionally, note that $\log f(U_{ij}|\theta )$ is concave. By Theorem 2.7 in Newey and McFadden [27], $\hat{\theta }\to_{p}\theta_{0}$.

### Appendix A.2. Proofs of Theorem 2

Condition (i) of Theorem 3.3 in Newey and McFadden [27] is satisfied by assumption (A1); (ii) is satisfied since the likelihood function is twice continuously differentiable; (iii) is satisfied since the moments and parameters are bounded by assumptions (A1) and (A2); (iv) is satisfied by assumption (A3). By differentiating (A2) twice, we obtain the hessian matrix shown below. The expectations of the moments and parameters are all bounded by assumptions (A1)–(A2) so condition (v) is satisfied. By Theorem 3.3 in Newey and McFadden, [27] we establish $\sqrt{n}\left(\hat{\theta }-\theta_{0}\right)\to_{D}N\left(0,{\left[I\left(\theta_{0}\right)\right]}^{-1}\right),$ where $I(\theta_{0})$ is the expected Fisher information at $\theta_{0}$.

The first partial derivatives of the log-likelihood are:

$$\begin{array}{c} \frac{\partial l(\theta )}{\partial \mu }=\sum_{j=1}^{g}\sum_{i=1}^{n_{j}}\left\{Y_{ij}\left(1+R_{ij}^{\prime }\delta \right)-\frac{\exp(W_{ij}^{\prime }\left(\delta \right)\beta^{*})}{1+\exp(W_{ij}^{\prime }\left(\delta \right)\beta^{*})}\left(1+R_{ij}^{\prime }\delta \right)\right\}, \end{array}$$

$$\begin{array}{c} \frac{\partial l(\theta )}{\partial \tau_{j}}=\sum_{i=1}^{n_{j}}\left\{Y_{ij}T_{i,j}\left(1+R_{ij}^{\prime }\delta \right)-\frac{\exp(W_{ij}^{\prime }\left(\delta \right)\beta^{*})}{1+\exp(W_{ij}^{\prime }\left(\delta \right)\beta^{*})}T_{i,j}\left(1+R_{ij}^{\prime }\delta \right)\right\}, \end{array}$$

$$\begin{array}{c} \frac{\partial l(\theta )}{\partial \beta_{k}}=\sum_{j=1}^{g}\sum_{i=1}^{n_{j}}\left\{Y_{ij}X_{ik}\left(1+R_{ij}^{\prime }\delta \right)-\frac{\exp(W_{ij}^{\prime }\left(\delta \right)\beta^{*})}{1+\exp(W_{ij}^{\prime }\left(\delta \right)\beta^{*})}X_{ik}\left(1+R_{ij}^{\prime }\delta \right)\right\}, \end{array}$$

$$\begin{array}{c} \frac{\partial l(\theta )}{\partial \delta_{j}}=\sum_{i=1}^{n_{j}}\left\{Y_{ij}V_{ij}^{\prime }\beta^{*}R_{i,j}-\frac{\exp(W_{ij}^{\prime }\left(\delta \right)\beta^{*})}{1+\exp(W_{ij}^{\prime }\left(\delta \right)\beta^{*})}V_{ij}^{\prime }\beta^{*}R_{i,j}\right\}, \end{array}$$

for j=1,2,...,g−1 and k=1,2,...,p.

The second partial derivatives of the log-likelihood are:

$$\begin{array}{c} \frac{\partial^{2}l\left(\theta \right)}{\partial \mu^{2}}=\sum_{j=1}^{g}\sum_{i=1}^{n_{j}}\left\{-\frac{\exp(W_{ij}^{\prime }\left(\delta \right)\beta^{*})}{{\left[1+\exp\left(W_{ij}^{\prime }\left(\delta \right)\beta^{*}\right)\right]}^{2}}{\left(1+R_{ij}^{\prime }\delta \right)}^{2}\right\}, \end{array}$$

$$\begin{array}{c} \frac{\partial^{2}l\left(\theta \right)}{\partial \mu \partial \tau_{j}}=\sum_{i=1}^{n_{j}}\left\{-\frac{\exp(W_{ij}^{\prime }\left(\delta \right)\beta^{*})}{{\left[1+\exp\left(W_{ij}^{\prime }\left(\delta \right)\beta^{*}\right)\right]}^{2}}T_{i,j}{\left(1+R_{ij}^{\prime }\delta \right)}^{2}\right\}, \end{array}$$

$$\begin{array}{c} \frac{\partial^{2}l\left(\theta \right)}{\partial \mu \beta_{k}}=\sum_{j=1}^{g}\sum_{i=1}^{n_{j}}\left\{-\frac{\exp(W_{ij}^{\prime }\left(\delta \right)\beta^{*})}{{\left[1+\exp\left(W_{ij}^{\prime }\left(\delta \right)\beta^{*}\right)\right]}^{2}}X_{ik}{\left(1+R_{ij}^{\prime }\delta \right)}^{2}\right\}, \end{array}$$

$$\begin{array}{c} \frac{\partial^{2}l\left(\theta \right)}{\partial \mu \partial \delta_{j}}=\sum_{i=1}^{n_{j}}\left\{Y_{ij}R_{i,j}-\left[\frac{V_{ij}^{\prime }\beta^{*}R_{i,j}\left(1+R_{ij}^{\prime }\delta \right)}{1+\exp(W_{ij}^{\prime }\left(\delta \right)\beta^{*})}+R_{i,j}\right]\frac{\exp(W_{ij}^{\prime }\left(\delta \right)\beta^{*})}{1+\exp(W_{ij}^{\prime }\left(\delta \right)\beta^{*})}\right\}, \end{array}$$

$$\begin{array}{c} \frac{\partial^{2}l\left(\theta \right)}{\partial \tau_{j}^{2}}=\sum_{i=1}^{n_{j}}\left\{-\frac{\exp(W_{ij}^{\prime }\left(\delta \right)\beta^{*})}{{\left[1+\exp\left(W_{ij}^{\prime }\left(\delta \right)\beta^{*}\right)\right]}^{2}}T_{i,j}^{2}{\left(1+R_{ij}^{\prime }\delta \right)}^{2}\right\}, \end{array}$$

$$\begin{array}{c} \frac{\partial^{2}l\left(\theta \right)}{\partial \tau_{j}\partial \beta_{k}}=\sum_{i=1}^{n_{j}}\left\{-\frac{\exp(W_{ij}^{\prime }\left(\delta \right)\beta^{*})}{{\left[1+\exp\left(W_{ij}^{\prime }\left(\delta \right)\beta^{*}\right)\right]}^{2}}T_{i,j}X_{ik}{\left(1+R_{ij}^{\prime }\delta \right)}^{2}\right\}, \end{array}$$

$$\begin{array}{c} \frac{\partial^{2}l\left(\theta \right)}{\partial \tau_{j}\partial \delta_{j}}=\sum_{i=1}^{n_{j}}\left\{Y_{ij}T_{i,j}R_{i,j}-\left[\frac{\left(V_{ij}^{\prime }\beta^{*}R_{i,j}\right)T_{i,j}\left(1+R_{ij}^{\prime }\delta \right)}{1+\exp(W_{ij}^{\prime }\left(\delta \right)\beta^{*})}+T_{i,j}R_{i,j}\right]\frac{\exp(W_{ij}^{\prime }\left(\delta \right)\beta^{*})}{1+\exp(W_{ij}^{\prime }\left(\delta \right)\beta^{*})}\right\}, \end{array}$$

$$\begin{array}{c} \frac{\partial l(\theta )}{\partial \beta_{k}\partial \beta_{k^{\prime }}}=\sum_{j=1}^{g}\sum_{i=1}^{n_{j}}\left\{-\frac{\exp(W_{ij}^{\prime }\left(\delta \right)\beta^{*})}{{\left[1+\exp\left(W_{ij}^{\prime }\left(\delta \right)\beta^{*}\right)\right]}^{2}}X_{ik}X_{ik^{\prime }}{\left(1+R_{ij}^{\prime }\delta \right)}^{2}\right\}, \end{array}$$

$$\begin{array}{c} \frac{\partial^{2}l\left(\theta \right)}{\partial \beta_{k}\partial \delta_{j}}=\sum_{i=1}^{n_{j}}\left\{Y_{ij}X_{ik}R_{i,j}-\left[\frac{\left(V_{ij}^{\prime }\beta^{*}R_{i,j}\right)X_{ik}\left(1+R_{ij}^{\prime }\delta \right)}{1+\exp(W_{ij}^{\prime }\left(\delta \right)\beta^{*})}+X_{ik}R_{i,j}\right]\frac{\exp(W_{ij}^{\prime }\left(\delta \right)\beta^{*})}{1+\exp(W_{ij}^{\prime }\left(\delta \right)\beta^{*})}\right\}, \end{array}$$

$$\begin{array}{c} \frac{\partial^{2}l\left(\theta \right)}{\partial \delta_{j}^{2}}=\sum_{i=1}^{n_{j}}\left\{-\frac{\exp(W_{ij}^{\prime }\left(\delta \right)\beta^{*})}{{\left[1+\exp\left(W_{ij}^{\prime }\left(\delta \right)\beta^{*}\right)\right]}^{2}}{\left(V_{ij}^{\prime }\beta^{*}R_{i,j}\right)}^{2}\right\}. \end{array}$$

The elements of the expected fisher information matrix at θ are easily obtained for terms without $Y_{ij}$. Here, we will only show the elements of the expected Fisher information matrix for terms involving $Y_{ij}$:

$$\begin{array}{c} -E\left(\frac{\partial^{2}l\left(\theta \right)}{\partial \mu \partial \delta_{j}}\right)=E\left\{\frac{\exp(W_{ij}^{\prime }\left(\delta \right)\beta^{*})}{{\left[1+\exp\left(W_{ij}^{\prime }\left(\delta \right)\beta^{*}\right)\right]}^{2}}V_{ij}^{\prime }\beta^{*}R_{i,j}\left(1+R_{ij}^{\prime }\delta \right)\right\}, \end{array}$$

$$\begin{array}{c} -E\left(\frac{\partial^{2}l\left(\theta \right)}{\partial \tau_{j}\partial \delta_{j}}\right)=E\left\{\frac{\exp(W_{ij}^{\prime }\left(\delta \right)\beta^{*})}{{\left[1+\exp\left(W_{ij}^{\prime }\left(\delta \right)\beta^{*}\right)\right]}^{2}}\left(V_{ij}^{\prime }\beta^{*}R_{i,j}\right)T_{i,j}\left(1+R_{ij}^{\prime }\delta \right)\right\}, \end{array}$$

$$\begin{array}{c} -E\left(\frac{\partial^{2}l\left(\theta \right)}{\partial \beta_{k}\partial \delta_{j}}\right)=E\left\{\frac{\exp(W_{ij}^{\prime }\left(\delta \right)\beta^{*})}{{\left[1+\exp\left(W_{ij}^{\prime }\left(\delta \right)\beta^{*}\right)\right]}^{2}}\left(V_{ij}^{\prime }\beta^{*}R_{i,j}\right)X_{ik}\left(1+R_{ij}^{\prime }\delta \right)\right\}, \end{array}$$

Using the notations defined in the main text $Z_{ij}={\left(W_{ij}^{\prime }\left(\delta_{0}\right),V_{ij}^{\prime }\beta_{0}^{*}R_{i,1},...,V_{ij}^{\prime }\beta_{0}^{*}R_{i,(g-1)}\right)}^{\prime }$ be a (2g+p−1)×1, we can rewrite the expected Fisher information in the following matrix form:

(A4) $$I\left(\theta \right)=E\{\phi \left(W_{ij}^{\prime }\left(\delta_{0}\right)\beta_{0}^{*}\right)\left[1-\phi \left(W_{ij}^{\prime }\left(\delta_{0}\right)\beta_{0}^{*}\right)\right]Z_{ij}Z_{ij}^{\prime }\}.$$

**Table A1:** Estimate, standard deviation (SD), standard error (SE), and coverage probability (CP) when $\tau_{1,0}=-1$ and $\delta_{1,0}=-0.5$ with 1000 simulation runs for the Absolute and Relative Treatment Effects model and logistic regression.

		Absolute and Relative Treatment Effects Model				Logistic Regression Model			
*n*	Parameter	Estimate	SD	SE	CP	Estimate	SD	SE	CP
300	δ	−0.476	0.236	0.232	0.944	-	-	-	-
	τ	−1.268	0.574	0.568	0.922	−0.533	0.190	0.182	0.270
	$\beta_{1}$	−0.516	0.186	0.182	0.954	−0.372	0.128	0.130	0.824
	$\beta_{1}$	0.534	0.188	0.184	0.948	0.385	0.130	0.131	0.844
	$\beta_{1}$	−0.508	0.179	0.181	0.957	−0.371	0.129	0.130	0.817
	$\beta_{1}$	0.517	0.190	0.183	0.944	0.374	0.136	0.131	0.818
500	δ	−0.479	0.189	0.175	0.943	-	-	-	-
	τ	−1.139	0.422	0.414	0.929	−0.549	0.146	0.140	0.118
	$\beta_{1}$	−0.509	0.144	0.139	0.951	−0.371	0.102	0.101	0.723
	$\beta_{2}$	0.504	0.140	0.136	0.947	0.368	0.101	0.009	0.729
	$\beta_{3}$	−0.509	0.148	0.139	0.938	−0.370	0.105	0.100	0.727
	$\beta_{4}$	0.510	0.141	0.138	0.942	0.371	0.101	0.100	0.741
700	δ	−0.484	0.153	0.145	0.943	-	-	-	-
	τ	−1.059	0.310	0.292	0.942	−0.529	0.119	0.118	0.025
	$\beta_{1}$	−0.502	0.119	0.116	0.940	−0.367	0.085	0.083	0.636
	$\beta_{2}$	0.502	0.113	0.116	0.953	0.369	0.080	0.084	0.634
	$\beta_{3}$	−0.507	0.125	0.116	0.934	−0.372	0.091	0.082	0.643
	$\beta_{4}$	0.511	0.121	0.117	0.944	0.372	0.088	0.083	0.633
1000	δ	−0.499	0.122	0.118	0.940	-	-	-	-
	τ	−1.061	0.241	0.221	0.946	−0.531	0.105	0.098	0.007
	$\beta_{1}$	−0.508	0.098	0.096	0.951	−0.369	0.070	0.069	0.522
	$\beta_{2}$	0.507	0.100	0.097	0.952	0.367	0.070	0.072	0.514
	$\beta_{3}$	−0.505	0.096	0.095	0.960	−0.366	0.069	0.071	0.520
	$\beta_{4}$	0.511	0.096	0.097	0.954	0.370	0.069	0.070	0.541

**Table A2:** Estimate, standard deviation (SD), standard error (SE), and coverage probability (CP) when $\tau_{1,0}=-1$ and $\delta_{1,0}=0.3$ with 1000 simulation runs for the Absolute and Relative Treatment Effects model and logistic regression.

		Absolute and Relative Treatment Effects Model				Logistic Regression Model			
*n*	Parameter	Estimate	SD	SE	CP	Estimate	SD	SE	CP
300	δ	0.340	0.346	0.411	0.938	-	-	-	-
	τ	−1.056	0.314	0.320	0.966	−1.255	0.224	0.218	0.797
	$\beta_{1}$	−0.516	0.154	0.155	0.958	−0.573	0.147	0.148	0.934
	$\beta_{2}$	0.527	0.155	0.157	0.956	0.588	0.149	0.149	0.922
	$\beta_{3}$	−0.516	0.148	0.155	0.967	−0.574	0.149	0.148	0.936
	$\beta_{4}$	0.522	0.156	0.156	0.953	0.580	0.153	0.149	0.924
500	δ	0.340	0.291	0.315	0.949	-	-	-	-
	τ	−1.043	0.237	0.241	0.968	−1.266	0.171	0.168	0.677
	$\beta_{1}$	−0.511	0.119	0.115	0.956	−0.572	0.116	0.113	0.904
	$\beta_{2}$	0.507	0.116	0.116	0.958	0.569	0.112	0.112	0.918
	$\beta_{3}$	−0.510	0.121	0.116	0.949	−0.571	0.116	0.114	0.922
	$\beta_{4}$	0.510	0.117	0.114	0.953	0.572	0.113	0.113	0.910
700	δ	0.330	0.242	0.263	0.956	-	-	-	-
	τ	−1.017	0.194	0.198	0.957	−1.242	0.137	0.140	0.596
	$\beta_{1}$	−0.505	0.102	0.098	0.947	−0.568	0.101	0.095	0.890
	$\beta_{2}$	0.506	0.095	0.099	0.956	0.568	0.092	0.096	0.906
	$\beta_{3}$	−0.506	0.105	0.097	0.941	−0.569	0.101	0.097	0.893
	$\beta_{4}$	0.509	0.100	0.100	0.954	0.574	0.099	0.096	0.881
1000	δ	0.312	0.212	0.216	0.955	-	-	-	-
	τ	−1.024	0.167	0.167	0.960	−1.245	0.113	0.117	0.438
	$\beta_{1}$	−0.508	0.083	0.084	0.946	−0.569	0.080	0.081	0.866
	$\beta_{2}$	0.507	0.084	0.083	0.954	0.569	0.080	0.079	0.867
	$\beta_{3}$	−0.507	0.083	0.083	0.951	−0.568	0.079	0.079	0.876
	$\beta_{4}$	0.508	0.080	0.082	0.947	0.570	0.077	0.080	0.881

**Table A3:** Estimate, standard deviation (SD), standard error (SE), and coverage probability (CP) when $\tau_{1,0}=-1$ and $\delta_{1,0}=0.5$ with 1000 simulation runs for the Absolute and Relative Treatment Effects model and logistic regression.

		Absolute and Relative Treatment Effects Model				Logistic Regression Model			
*n*	Parameter	Estimate	SD	SE	CP	Estimate	SD	SE	CP
300	δ	0.530	0.338	0.455	0.946	-	-	-	-
	τ	−1.053	0.272	0.298	0.981	−1.394	0.228	0.227	0.625
	$\beta_{1}$	−0.519	0.148	0.151	0.963	−0.612	0.154	0.151	0.897
	$\beta_{2}$	0.528	0.148	0.153	0.965	0.625	0.154	0.152	0.891
	$\beta_{3}$	−0.519	0.144	0.150	0.964	−0.611	0.154	0.152	0.906
	$\beta_{4}$	0.525	0.150	0.152	0.958	0.619	0.160	0.153	0.887
500	δ	0.527	0.292	0.348	0.948	-	-	-	-
	τ	−1.047	0.214	0.228	0.975	−1.404	0.171	0.175	0.344
	$\beta_{1}$	−0.513	0.113	0.114	0.962	−0.609	0.118	0.116	0.862
	$\beta_{2}$	0.510	0.110	0.112	0.960	0.606	0.114	0.117	0.876
	$\beta_{3}$	−0.513	0.115	0.112	0.959	−0.608	0.118	0.115	0.860
	$\beta_{4}$	0.514	0.113	0.113	0.962	0.609	0.117	0.117	0.865
700	δ	0.536	0.265	0.297	0.952	-	-	-	-
	τ	−1.016	0.181	0.188	0.943	−1.379	0.138	0.146	0.222
	$\beta_{1}$	−0.505	0.098	0.096	0.946	−0.604	0.103	0.097	0.819
	$\beta_{2}$	0.506	0.092	0.096	0.960	0.604	0.095	0.097	0.826
	$\beta_{3}$	−0.508	0.103	0.097	0.942	−0.607	0.105	0.098	0.811
	$\beta_{4}$	0.509	0.096	0.097	0.952	0.610	0.101	0.098	0.794
1000	δ	0.511	0.235	0.244	0.956	-	-	-	-
	τ	−1.022	0.156	0.158	0.961	−1.379	0.114	0.122	0.102
	$\beta_{1}$	−0.507	0.080	0.082	0.954	−0.605	0.082	0.084	0.765
	$\beta_{2}$	0.508	0.082	0.081	0.954	0.605	0.083	0.083	0.761
	$\beta_{3}$	−0.507	0.080	0.080	0.953	−0.604	0.081	0.084	0.764
	$\beta_{4}$	0.508	0.078	0.081	0.950	0.606	0.080	0.082	0.759

**Table A4:** Estimate, standard deviation (SD), standard error (SE), and coverage probability (CP) when $\tau_{1,0}=0$ and $\delta_{1,0}=-0.5$ with 1000 simulation runs for the Absolute and Relative Treatment Effects model and logistic regression.

		Absolute and Relative Treatment Effects Model				Logistic Regression Model			
*n*	Parameter	Estimate	SD	SE	CP	Estimate	SD	SE	CP
300	δ	−0.478	0.236	0.227	0.939	-	-	-	-
	τ	0.034	0.550	0.545	0.943	0.008	0.184	0.176	0.943
	$\beta_{1}$	−0.518	0.187	0.181	0.950	−0.373	0.131	0.128	0.819
	$\beta_{2}$	0.528	0.189	0.183	0.952	0.375	0.133	0.129	0.810
	$\beta_{3}$	−0.508	0.179	0.181	0.957	−0.367	0.128	0.128	0.804
	$\beta_{4}$	0.519	0.187	0.182	0.955	0.372	0.132	0.129	0.809
500	δ	−0.481	0.184	0.171	0.939	-	-	-	-
	τ	−0.008	0.317	0.307	0.947	−0.005	0.147	0.135	0.930
	$\beta_{1}$	−0.508	0.140	0.138	0.951	−0.367	0.100	0.099	0.704
	$\beta_{2}$	0.506	0.138	0.136	0.948	0.366	0.098	0.098	0.731
	$\beta_{3}$	−0.510	0.145	0.137	0.940	−0.369	0.099	0.099	0.725
	$\beta_{4}$	0.509	0.142	0.138	0.945	0.366	0.100	0.099	0.715
700	δ	-0.482	0.143	0.143	0.956	-	-	-	-
	τ	−0.009	0.237	0.240	0.954	−0.004	0.117	0.114	0.940
	$\beta_{1}$	−0.503	0.119	0.116	0.943	−0.367	0.085	0.082	0.613
	$\beta_{2}$	0.501	0.113	0.115	0.956	0.366	0.081	0.081	0.628
	$\beta_{3}$	−0.507	0.123	0.115	0.946	−0.369	0.090	0.082	0.614
	$\beta_{4}$	0.512	0.118	0.116	0.949	0.371	0.084	0.083	0.636
1000	δ	−0.500	0.118	0.116	0.956	-	-	-	-
	τ	−0.005	0.205	0.200	0.950	−0.003	0.103	0.095	0.933
	$\beta_{1}$	−0.507	0.099	0.095	0.947	−0.366	0.068	0.070	0.497
	$\beta_{2}$	0.509	0.100	0.098	0.949	0.366	0.070	0.069	0.500
	$\beta_{3}$	−0.506	0.094	0.097	0.951	−0.363	0.068	0.068	0.492
	$\beta_{4}$	0.509	0.099	0.097	0.950	0.365	0.071	0.069	0.506

**Table A5:** Estimate, standard deviation (SD), standard error (SE), and coverage probability (CP) when $\tau_{1,0}=0$ and $\delta_{1,0}=-0.3$ with 1000 simulation runs for the Absolute and Relative Treatment Effects model and logistic regression.

		Absolute and Relative Treatment Effects Model				Logistic Regression Model			
*n*	Parameter	Estimate	SD	SE	CP	Estimate	SD	SE	CP
300	δ	−0.264	0.269	0.266	0.933	-	-	-	-
	τ	0.013	0.293	0.289	0.946	0.007	0.184	0.179	0.942
	$\beta_{1}$	−0.519	0.175	0.172	0.947	−0.434	0.133	0.133	0.920
	$\beta_{2}$	0.524	0.178	0.174	0.957	0.435	0.136	0.132	0.903
	$\beta_{3}$	−0.509	0.170	0.171	0.955	−0.425	0.130	0.132	0.901
	$\beta_{4}$	0.518	0.176	0.173	0.958	0.431	0.134	0.132	0.908
500	δ	−0.273	0.211	0.201	0.932	-	-	-	-
	τ	−0.011	0.217	0.208	0.941	−0.007	0.148	0.137	0.935
	$\beta_{1}$	−0.508	0.134	0.131	0.948	−0.426	0.104	0.102	0.875
	$\beta_{2}$	0.506	0.130	0.130	0.944	0.425	0.099	0.101	0.885
	$\beta_{3}$	−0.509	0.136	0.132	0.942	−0.426	0.101	0.103	0.894
	$\beta_{4}$	0.508	0.134	0.131	0.949	0.425	0.101	0.101	0.876
700	δ	−0.282	0.169	0.167	0.949	-	-	-	-
	τ	−0.003	0.168	0.170	0.956	−0.002	0.118	0.116	0.945
	$\beta_{1}$	−0.503	0.112	0.111	0.945	−0.423	0.087	0.084	0.832
	$\beta_{2}$	0.500	0.107	0.111	0.954	0.420	0.082	0.083	0.846
	$\beta_{3}$	−0.506	0.117	0.110	0.942	−0.425	0.091	0.085	0.833
	$\beta_{4}$	0.511	0.111	0.110	0.950	0.428	0.085	0.085	0.845
1000	δ	−0.297	0.141	0.136	0.951	-	-	-	-
	τ	−0.004	0.146	0.142	0.952	−0.003	0.101	0.097	0.942
	$\beta_{1}$	−0.507	0.094	0.091	0.947	−0.424	0.071	0.070	0.797
	$\beta_{2}$	0.508	0.095	0.092	0.950	0.424	0.071	0.073	0.798
	$\beta_{3}$	−0.506	0.089	0.093	0.953	−0.421	0.070	0.072	0.795
	$\beta_{4}$	0.508	0.095	0.092	0.941	0.423	0.072	0.071	0.787

**Table A6:** Estimate, standard deviation (SD), standard error (SE), and coverage probability (CP) when $\tau_{1,0}=0$ and $\delta_{1,0}=0$ with 1000 simulation runs for the Absolute and Relative Treatment Effects model and logistic regression.

		Absolute and Relative Treatment Effects Model				Logistic Regression Model			
*n*	Parameter	Estimate	SD	SE	CP	Estimate	SD	SE	CP
300	δ	0.038	0.295	0.327	0.937	-	-	-	-
	τ	0.006	0.204	0.199	0.941	0.004	0.185	0.183	0.941
	$\beta_{1}$	−0.521	0.165	0.159	0.943	−0.515	0.141	0.139	0.951
	$\beta_{2}$	0.524	0.161	0.162	0.957	0.517	0.138	0.137	0.965
	$\beta_{3}$	−0.512	0.158	0.159	0.952	−0.507	0.137	0.137	0.954
	$\beta_{4}$	0.518	0.162	0.161	0.956	0.512	0.139	0.137	0.951
500	δ	0.036	0.248	0.251	0.936	-	-	-	-
	τ	−0.005	0.154	0.147	0.951	−0.005	0.148	0.141	0.934
	$\beta_{1}$	−0.510	0.122	0.123	0.955	−0.508	0.106	0.104	0.957
	$\beta_{2}$	0.506	0.121	0.122	0.947	0.505	0.102	0.105	0.957
	$\beta_{3}$	−0.508	0.125	0.121	0.947	−0.506	0.105	0.102	0.955
	$\beta_{4}$	0.507	0.125	0.122	0.941	0.505	0.107	0.105	0.943
700	δ	0.026	0.211	0.209	0.952	-	-	-	-
	τ	−0.002	0.121	0.122	0.956	−0.002	0.119	0.118	0.954
	$\beta_{1}$	−0.504	0.104	0.103	0.952	−0.502	0.090	0.089	0.947
	$\beta_{2}$	0.501	0.099	0.102	0.957	0.500	0.085	0.087	0.959
	$\beta_{3}$	−0.506	0.108	0.102	0.943	−0.505	0.093	0.088	0.935
	$\beta_{4}$	0.510	0.103	0.103	0.949	0.509	0.087	0.089	0.952
1000	δ	0.008	0.178	0.171	0.944	-	-	-	-
	τ	−0.001	0.105	0.102	0.955	−0.002	0.102	0.099	0.944
	$\beta_{1}$	−0.507	0.088	0.087	0.953	−0.503	0.074	0.075	0.956
	$\beta_{2}$	0.508	0.088	0.086	0.947	0.505	0.073	0.074	0.951
	$\beta_{3}$	−0.505	0.083	0.085	0.957	−0.501	0.071	0.072	0.962
	$\beta_{4}$	0.508	0.087	0.084	0.951	0.504	0.075	0.074	0.949

**Table A7:** Estimate, standard deviation (SD), standard error (SE), and coverage probability (CP) when $\tau_{1,0}=-1$ and $\delta_{1,0}=-0.3$ with 1000 simulation runs for the Absolute and Relative Treatment Effects model and logistic regression with treatment effects, covariate effects, and interaction effects.

		Absolute and Relative Treatment Effects Model				Logistic Regression Model			
*n*	Parameter	Estimate	SD	SE	CP	Estimate	SD	SE	CP
300	δ	−0.310	0.147	0.145	0.951	-	-	-	-
	τ	−1.031	0.145	0.143	0.948	−1.035	0.144	0.141	0.950
	$\beta_{1}$	−0.549	0.235	0.217	0.962	−0.400	0.157	0.145	0.869
	$\beta_{2}$	0.532	0.213	0.214	0.963	0.387	0.147	0.146	0.881
	$\beta_{3}$	−0.545	0.226	0.217	0.968	−0.395	0.152	0.147	0.868
	$\beta_{4}$	0.538	0.213	0.214	0.963	0.393	0.147	0.145	0.868
500	δ	−0.310	0.129	0.126	0.947	-	-	-	-
	τ	−1.016	0.124	0.123	0.950	−1.022	0.124	0.122	0.947
	$\beta_{1}$	−0.540	0.179	0.175	0.973	−0.398	0.124	0.125	0.850
	$\beta_{2}$	0.532	0.190	0.179	0.966	0.392	0.129	0.123	0.844
	$\beta_{3}$	−0.536	0.180	0.177	0.959	−0.394	0.127	0.124	0.832
	$\beta_{4}$	0.534	0.197	0.179	0.946	0.394	0.138	0.126	0.828
700	δ	−0.300	0.086	0.088	0.958	-	-	-	-
	τ	−1.011	0.084	0.086	0.950	−1.021	0.086	0.085	0.944
	$\beta_{1}$	−0.511	0.119	0.116	0.948	−0.389	0.092	0.087	0.741
	$\beta_{2}$	0.518	0.117	0.117	0.965	0.395	0.085	0.088	0.751
	$\beta_{3}$	−0.515	0.116	0.116	0.963	−0.394	0.088	0.087	0.756
	$\beta_{4}$	0.518	0.119	0.117	0.966	0.396	0.087	0.088	0.769
1000	δ	−0.305	0.076	0.079	0.957	-	-	-	-
	τ	−1.004	0.076	0.076	0.958	−1.014	0.077	0.076	0.951
	$\beta_{1}$	−0.512	0.105	0.103	0.959	−0.389	0.080	0.077	0.676
	$\beta_{2}$	0.517	0.103	0.105	0.970	0.394	0.078	0.075	0.723
	$\beta_{3}$	−0.513	0.101	0.104	0.969	−0.390	0.075	0.076	0.714
	$\beta_{4}$	0.516	0.103	0.104	0.964	0.393	0.079	0.078	0.696

**Table A8:** Estimate, standard deviation (SD), standard error (SE), and coverage probability (CP) when $\tau_{1,0}=-1$ and $\delta_{1,0}=0$ with 1000 simulation runs for the Absolute and Relative Treatment Effects model and logistic regression with treatment effects, covariate effects, and interaction effects.

		Absolute and Relative Treatment Effects Model				Logistic Regression Model			
*n*	Parameter	Estimate	SD	SE	CP	Estimate	SD	SE	CP
300	δ	−0.003	0.143	0.146	0.953	-	-	-	-
	τ	−1.033	0.150	0.148	0.946	−1.025	0.145	0.145	0.949
	$\beta_{1}$	−0.532	0.172	0.167	0.950	−0.519	0.160	0.153	0.943
	$\beta_{2}$	0.529	0.171	0.168	0.951	0.516	0.159	0.152	0.942
	$\beta_{3}$	−0.531	0.169	0.169	0.961	−0.519	0.154	0.154	0.954
	$\beta_{4}$	0.528	0.168	0.168	0.963	0.516	0.154	0.153	0.953
500	δ	−0.004	0.130	0.126	0.945	-	-	-	-
	τ	−1.019	0.126	0.127	0.945	−1.014	0.123	0.124	0.947
	$\beta_{1}$	−0.526	0.141	0.142	0.954	−0.515	0.128	0.132	0.959
	$\beta_{2}$	0.516	0.145	0.144	0.954	0.506	0.133	0.130	0.948
	$\beta_{3}$	−0.525	0.146	0.144	0.953	−0.514	0.133	0.131	0.952
	$\beta_{4}$	0.523	0.153	0.143	0.944	0.513	0.141	0.131	0.929
700	δ	0.001	0.087	0.088	0.961	-	-	-	-
	τ	−1.012	0.088	0.089	0.957	−1.009	0.087	0.087	0.952
	$\beta_{1}$	−0.508	0.098	0.096	0.951	−0.504	0.094	0.091	0.938
	$\beta_{2}$	0.512	0.094	0.099	0.953	0.509	0.089	0.092	0.957
	$\beta_{3}$	−0.512	0.097	0.097	0.954	−0.509	0.091	0.093	0.954
	$\beta_{4}$	0.514	0.098	0.098	0.947	0.510	0.092	0.092	0.947
1000	δ	−0.001	0.077	0.079	0.957	-	-	-	-
	τ	−1.004	0.080	0.079	0.948	−1.002	0.079	0.078	0.948
	$\beta_{1}$	−0.507	0.088	0.086	0.949	−0.504	0.083	0.081	0.953
	$\beta_{2}$	0.511	0.088	0.087	0.959	0.508	0.083	0.080	0.954
	$\beta_{3}$	−0.505	0.082	0.086	0.958	−0.502	0.077	0.082	0.965
	$\beta_{4}$	0.508	0.086	0.087	0.960	0.505	0.081	0.082	0.959

**Table A9:** Estimate, standard deviation (SD), standard error (SE), and coverage probability (CP) when $\tau_{1,0}=-1$ and $\delta_{1,0}=0.3$ with 1000 simulation runs for the Absolute and Relative Treatment Effects model and logistic regression with treatment effects, covariate effects, and interaction effects.

		Absolute and Relative Treatment Effects Model				Logistic Regression Model			
*n*	Parameter	Estimate	SD	SE	CP	Estimate	SD	SE	CP
300	δ	0.302	0.149	0.146	0.944	-	-	-	-
	τ	−1.034	0.161	0.160	0.952	−0.938	0.140	0.143	0.929
	$\beta_{1}$	−0.528	0.183	0.181	0.952	−0.592	0.158	0.159	0.929
	$\beta_{2}$	0.514	0.178	0.180	0.958	0.576	0.156	0.157	0.943
	$\beta_{3}$	−0.520	0.182	0.179	0.962	−0.586	0.155	0.158	0.925
	$\beta_{4}$	0.517	0.184	0.180	0.951	0.581	0.156	0.157	0.932
500	δ	0.302	0.132	0.126	0.945	-	-	-	-
	τ	−1.026	0.138	0.138	0.945	−0.932	0.124	0.124	0.907
	$\beta_{1}$	−0.523	0.151	0.153	0.962	−0.591	0.131	0.137	0.920
	$\beta_{2}$	0.514	0.154	0.155	0.962	0.581	0.134	0.137	0.920
	$\beta_{3}$	−0.523	0.155	0.151	0.945	−0.590	0.136	0.134	0.918
	$\beta_{4}$	0.522	0.161	0.152	0.938	0.587	0.141	0.135	0.903
700	δ	0.303	0.086	0.089	0.960	-	-	-	-
	τ	−1.013	0.094	0.096	0.956	−0.923	0.084	0.087	0.866
	$\beta_{1}$	−0.505	0.104	0.104	0.952	−0.576	0.092	0.094	0.892
	$\beta_{2}$	0.510	0.101	0.103	0.963	0.581	0.091	0.095	0.883
	$\beta_{3}$	−0.512	0.104	0.103	0.953	−0.582	0.093	0.094	0.878
	$\beta_{4}$	0.512	0.105	0.105	0.952	0.583	0.094	0.095	0.878
1000	δ	0.300	0.076	0.079	0.967	-	-	-	-
	τ	−1.005	0.087	0.085	0.943	−0.918	0.078	0.077	0.798
	$\beta_{1}$	−0.502	0.094	0.091	0.948	−0.573	0.084	0.083	0.868
	$\beta_{2}$	0.507	0.093	0.092	0.949	0.577	0.083	0.083	0.859
	$\beta_{3}$	−0.503	0.090	0.091	0.954	−0.573	0.081	0.085	0.882
	$\beta_{4}$	0.504	0.091	0.092	0.959	0.574	0.082	0.084	0.871

## References

[1] Zhou X., Liu K.Y., Wong S. (2004). Cancer classification and prediction using logistic regression with Bayesian gene selection. J. Biomed. Inform..

[2] Lynam A., Dennis J., Owen K., Oram R.A., Jones A.G., Shields B.M., Ferrat L.A. (2020). Logistic regression has similar performance to optimised machine learning algorithms in a clinical setting: Application to the discrimination between type 1 and type 2 diabetes in young adults. Diagn. Progn. Res..

[3] Liem Y., Judge A., Kirwan J., Ourradi K., Li Y., Sharif M. (2020). Multivariable logistic and linear regression models for identification of clinically useful biomarkers for osteoarthritis. Sci. Rep. Vol..

[4] Cortes C., Vapnik V. (1995). Support-vector networks. Mach. Learn..

[5] Hastie T., Tibshirani R., Friedman J. (2001). The Elements of Statistical Learning: Data Mining, Inference, and Prediction.

[6] Harrison R., Kennedy R. (2005). Artificial neural network models for prediction of acute coronary syndromes using clinical data from the time of presentation. Ann. Emerg. Med..

[7] Frizzell J.D., Liang L., Schulte P.J., Yancy C.W., Heidenreich P.A., Hernandez A.F., Bhatt D.L., Fonarow G.C., Laskey W.K. (2017). Prediction of 30-day all-cause readmissions in patients hospitalized for heart failure: Comparison of machine learning and other statistical approaches. J. Am. Med. Assoc..

[8] Hsieh M.H., Sun L.M., Lin C.L., Hsieh M.J., Hsu C.Y., Kao C.H. (2018). Development of a prediction model for pancreatic cancer in patients with type 2 diabetes using logistic regression and artificial neural network models. Cancer Manag. Res..

[9] Kharde V., Sonawane S. (2016). Sentiment Analysis of Twitter Data: A Survey of Techniques. Int. J. Comput. Appl..

[10] Zhang Z. (2021). Five Critical Genes Related to Seven COVID-19 Subtypes: A Data Science Discovery. J. Data Sci..

[11] Greenland S., Schwartzbaum J., Finkle W. (2000). Problems Due to Small Samples and Sparse Data in Conditional Logistic Regression Analysis. Am. J. Epidemiol..

[12] Hadjcostas P. (2003). Consistency of logistic regression coefficient estimates calculated from a training sample. Stat. Probab. Lett..

[13] Tibshirani R. (1996). Regression shrinkage and selection via the lasso. J. R. Stat. Soc. Ser. B (Stat. Methodol.).

[14] Fan J., Li R. (2001). Variable selection via nonconcave penalized likelihood and its oracle properties. J. Am. Stat. Assoc..

[15] Zhang C.H. (2010). Nearly unbiased variable selection under minimax concave penalty. Ann. Stat..

[16] Tom B., Su L., Farewell V. (2016). A corrected formulation for marginal inference derived from two-part mixed models for longitudinal semi-continuous data. Stat. Methods Med. Res..

[17] Farewell V., Long D., Tom B., Yiu S., Su L. (2017). Two-Part and Related Regression Models for Longitudinal Data. Annu. Rev. Stat. Its Appl..

[18] Xie Y., Zhang Z., Rathouz P., Barrett B. (2018). Multivariate semi-continuous proportionally constrained two-part fixed effects models and applications. Stat. Methods Med. Res..

[19] Thaver D., Zaidi A. (2009). Burden of neonatal infections in developing countries: A review of evidence from community-based studies. Pediatr. Infect. Dis. J..

[20] Panigrahi P., Parida S., Nanda N., Satpathy R., Pradhan L., Chandel D.S., Baccaglini L., Mohapatra A., Mohapatra S.S., Misra P.R. (2017). A randomized synbiotic trial to prevent sepsis among infants in rural India. Nature.

[21] Barrett B., Hayney M., Muller D., Rakel D., Brown R., Zgierska A., Barlow S., Hayer S., Barnet J.H., Torres E.R. (2018). Meditation or exercise for preventing acute respiratory infection (MEPARI-2): A randomized controlled trial. PLoS ONE.

[22] Tsang T., Vicky J., Ip D., Perera R.A., So H.C., Leung G.M., Peiris J.M., Cowling B.J., Cauchemez S. (2019). Indirect protection from vaccinating children against influenza in households. Nat. Commun..

[23] Boulware D., Pullen M., Bangdiwala A., Pastick K.A., Lofgren S.M., Okafor E.C., Skipper C.P., Nascene A.A., Nicol M.R., Abassi M. (2020). A randomized trial of hydroxychloroquine as postexposure prophylaxis for COVID-19. N. Engl. J. Med..

[24] Lecronier M., Beurton A., Burrel S., Haudebourg L., Deleris R., Le Marec J., Virolle S., Nemlaghi S., Bureau C., Mora P. (2020). Comparison of hydroxychloroquine, lopinavir/ ritonavir, and standard of care in critically ill patients with SARS-CoV-2 pneumonia. Crit. Care.

[25] The Recovery Collaborative Group (2020). Effect of hydroxychloroquine in hospitalized patients with COVID-19. N. Engl. J. Med..

[26] WHO Solidarity Trial Consortium (2021). Repurposed antiviral drugs for COVID-19—Interim WHO Solidarity trial results. N. Engl. J. Med..

[27] Newey W., McFadden D. (1994). Large Sample Estimation and Hypothesis Testing. Handb. Econom..

